# Wearable Devices in Remote Cardiac Rehabilitation With and Without Weekly Online Coaching for Patients With Coronary Artery Disease: Randomized Controlled Trial

**DOI:** 10.2196/63797

**Published:** 2025-05-12

**Authors:** Ryota Nishio, Tomotaka Dohi, Miho Yokoyama, Taisuke Nakade, Norihito Takahashi, Yuichi Chikata, Hirohisa Endo, Hiroki Nishiyama, Iwao Okai, Hiroshi Iwata, Shinya Okazaki, Katsumi Miyauchi, Hiroyuki Daida, Tohru Minamino

**Affiliations:** 1 Department of Cardiovascular Biology and Medicine, Juntendo University Graduate School of Medicine, 2-1-1 Hongo, Bunkyo-ku, Tokyo, 113-0033, Japan, 81 3 3813 3111, 81 3 5802 3946; 2 Department of Cardiovascular Medicine, Juntendo University Shizuoka Hospital, Izunokuni, Shizuoka, Japan; 3 Japan Agency for Medical Research and Development, Japan Agency for Medical Research and Development-Core Research for Evolutionary Medical Science and Technology (AMED-CREST), Tokyo, Japan

**Keywords:** cardiac rehabilitation, cardiopulmonary exercise test, coronary artery disease, wearable device, wearable, cardiopulmonary, artery, rehabilitation, exercise, randomized trial, online coaching, cardiovascular, exercise program, home exercise program, monitoring system, monitor, anxiety, quality of life, VO_2_, anaerobic, coaching, mobile phone, oxygen consumption

## Abstract

**Background:**

Cardiac rehabilitation (CR) is effective in preventing cardiovascular diseases; however, participation in CR programs remains limited due to the associated challenges. The integration of wearable devices and real-time monitoring offers a potential solution to enhance adherence to remote CR programs and their outcomes.

**Objective:**

This study aimed to evaluate the efficacy of a remote CR program using wearable devices and real-time monitoring with or without online coaching (OLC) in improving exercise capacity in patients with coronary artery disease (CAD).

**Methods:**

We enrolled 50 patients with CAD in a remote CR program in this randomized, open-label, single-center pilot trial (phase III). After baseline cardiopulmonary exercise tests (CPETs), all patients were assigned a CPET-based home exercise program and were provided with a wearable device (Fitbit Sense; Fitbit, Inc) with a real-time monitoring system (Recoval; SapplyM, Inc). The patients were randomly assigned to an intervention group with OLC (n=25) or a control wearable device (CON; n=25) group. The primary outcomes were changes in peak oxygen consumption (peak VO_2_) and anaerobic threshold VO_2_ (oxygen consumption) at 12 weeks. The secondary outcomes were changes in CPET parameters, daily activity, anxiety levels, and health-related quality of life.

**Results:**

Peak VO_2_ and anaerobic threshold VO_2_ increased significantly from baseline to 12 weeks in the OLC (+1.6 mL/kg/min, *P*<.001; +1.0 mL/kg/min, *P*=.001) and CON (+0.6 mL/kg/min, *P*=.008; +1.3 mL/kg/min, *P*=.002) groups with no significant between-group differences (*P*=.65 and *P*=.90). In the latter half of the intervention, the daily distance and highly active time in the OLC group were significantly increased compared with those in the CON group (all *P*<.05). Mental status and health-related quality of life scores showed no significant differences between the groups. No major adverse cardiac events were reported. One patient in the OLC group dropped out due to lower limb muscle strain.

**Conclusions:**

Remote CR using wearable devices and a real-time monitoring system significantly improved exercise capacity in patients with CAD over a 12-week intervention program. The addition of regular OLC to the intervention program further enhanced the physical activity levels, particularly in high-intensity activities. Future studies are warranted to validate these findings in more diverse populations and over longer intervention periods to optimize the benefits and safety of remote CR programs.

## Introduction

Cardiac rehabilitation (CR) is beneficial not only in the aftermath of acute myocardial infarction but also in patients with coronary artery disease (CAD) after percutaneous coronary intervention for prior myocardial infarction, unstable angina, stable angina, and coronary artery bypass graft surgery [1-3]. Despite its numerous benefits, CR use remains insufficient, with various contributing factors such as limited access to rehabilitation facilities, financial constraints, busy schedules, and insufficient patient education [4,5]. A meta-analysis evaluating interventions to enhance adherence found that unmonitored CR and reduced copayments significantly improved adherence [6]. Recently, several studies have reported a negative correlation between cardiovascular disease risk and physical activity measured using wearable devices and a smartphone [7-9]. Furthermore, smartphone-based home CR programs have been shown to enhance participation and adherence and improve both physiological and psychological health outcomes [10].

Recent meta-analyses reported that wearable device-based interventions effectively increase daily activity levels and physical capacity in cardiovascular patients, especially when paired with feedback mechanisms [11]. However, there remains considerable heterogeneity with regard to the methods, frequency, and quality of feedback used in these wearable device-based interventions. For instance, while some studies have used weekly feedback sessions, others have implemented daily or real-time feedback, reflecting variability in intervention designs [12-15]. Additionally, the specific content and personalization of feedback have been underexplored, further highlighting the need for a standardized framework. Therefore, to address the limitations and gaps in prior research, this study aimed to provide insights into the most effective strategies for wearable device-based CR interventions. In this study, we developed a system for real-time monitoring of patient activity levels by integrating wearable devices and smartphones (Recoval; SapplyM, Inc). This system combines real-time data collection on physical activity and biometric metrics with online coaching (OLC) to deliver personalized feedback and support to the users. The primary objectives of this pilot study were to evaluate the efficacy of this system in improving exercise capacity and adherence through continuous and interactive communication between health care providers and patients and to clarify the synergistic effects of OLC on the use of wearable devices. We hypothesized that the combination of wearable devices and OLC would result in greater improvements in exercise capacity and adherence than those achieved through the use of wearable devices alone.

## Methods

### Eligibility Criteria

We enrolled 50 patients with CAD who were eligible for outpatient CR (phase III) for the secondary prevention of CAD at Juntendo University Hospital between April 30, 2022, and January 21, 2023. The exclusion criteria were as follows: (1) implantable medical device users such as patients with cardiac pacemakers and defibrillators; (2) patients diagnosed with acute myocardial infarction, uncontrolled angina pectoris not stabilized by medical treatment, uncontrolled arrhythmias causing subjective symptoms or hemodynamic abnormalities, uncontrolled heart failure, symptomatic severe aortic stenosis, or a psychiatric disorder impeding communication; and (3) participants deemed unsuitable for this study by the principal investigator and research physician.

### Study Design

**Figure 1.**
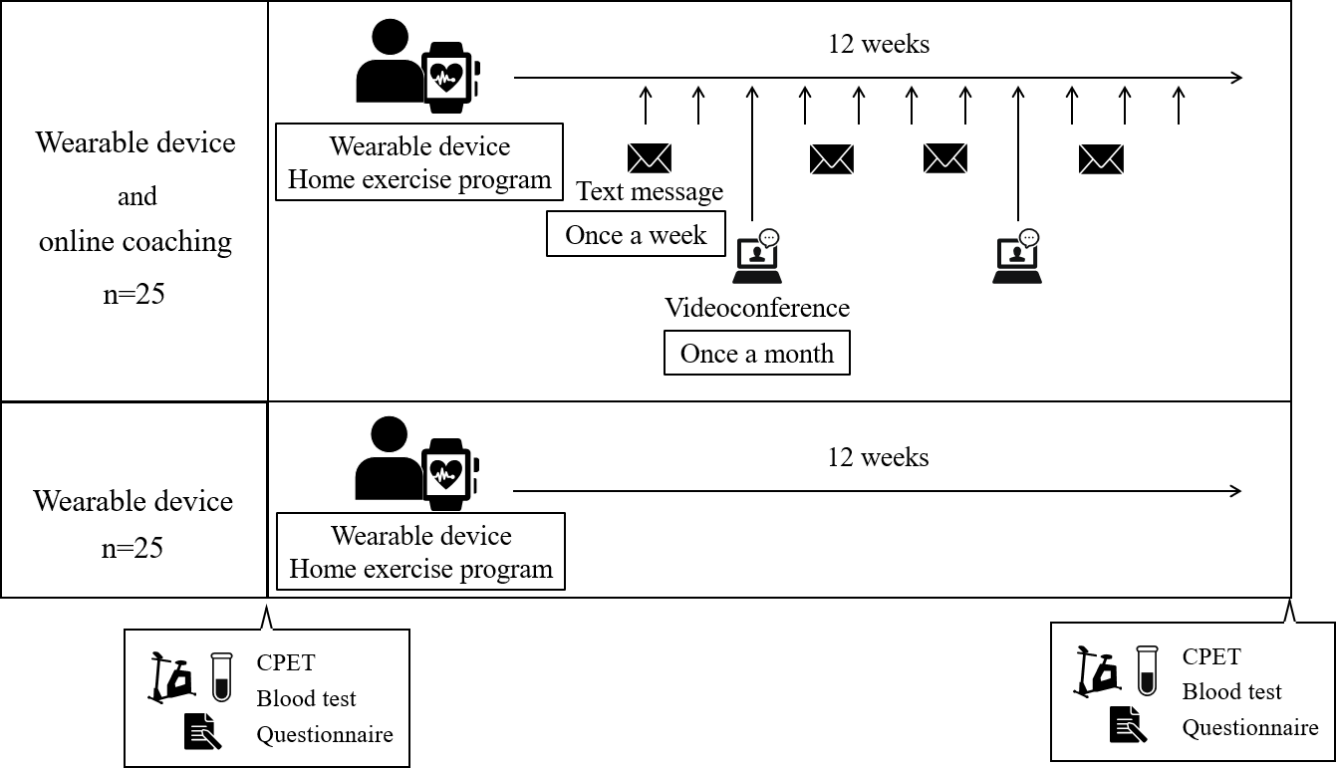
Timeline of this study. The patients were divided into the wearable device and online coaching group and the wearable device group. The intervention lasted for 12 weeks, and during this time, all patients were given a home exercise program. The wearable device and online coaching group received exercise guidance based on their activity levels recorded by their wearable device. This guidance was given through weekly text messages and monthly videoconferences. The wearable device group only wore the wearable device and did not receive any additional instruction. CPET: cardiopulmonary exercise test.

This was a randomized, open-label, comparative, parallel-group, single-center interventional study. This study followed the CONSORT (Consolidated Standards of Reporting Trials) reporting guidelines. In this study, patients were randomly divided into a wearable device plus OLC group and a control wearable device (CON) group, stratified by age (<60 years and ≥60 years) and sex (male and female), using a stratified permuted block design as the allocation method. The smartphones used by the patients were personal devices, and the wearable device was a Fitbit Sense. Patient data were collected using a wearable device linked to a smartphone for real-time monitoring of step count, activity level, and heart rate (Recoval; for detailed information about the product, please see [16]). A proprietary algorithm converted the acceleration signals from a wearable device into step counts and metabolic-equivalent tasks (METs). The intervention lasted for 12 weeks, and all patients underwent a cardiopulmonary exercise test (CPET)-based home exercise program. This program aims to engage in exercise at anaerobic threshold (AT) heart rate and a Borg scale of 12‐13, for at least 30 minutes per day, 5 days a week. Additionally, the patients in the OLC group were provided with exercise guidance based on their activity levels recorded by their wearable device. Guidance was provided via weekly text messages and monthly videoconferences. The participants in the CON group wore only the wearable device and did not receive any additional instructions (Figure 1).

### Ethical Considerations

The Ethics Committee of the Juntendo Clinical Research and Trial Centre approved this study (IRB number E21-0353). Patients were enrolled by outpatient primary care physicians who were not involved in the analysis, and external clinical research coordinators allocated them into the 2 groups. All patients provided written informed consent to participate in this study and had the option to opt out of this study at their discretion. The collected data were deidentified and managed accordingly. Additionally, no compensation was provided to study participants.

### Data Collection

#### Overview

Blood samples were collected and blood pressure (BP) was measured for all the participants at the beginning and end of this study. Patients with BP >140/90 mm Hg or those taking antihypertensive medications were considered hypertensive. Dyslipidemia was defined based on the values of low-density lipoprotein cholesterol, high-density lipoprotein cholesterol, and triglycerides, (≥140, ≤40, and ≥150 mg/dL, respectively) or use of statins or lipid-lowering agents [17]. Diabetes mellitus was defined as either a hemoglobin A_1c_ level of ≥6.5% or the use of medication with insulin or oral hypoglycemic drugs. Chronic kidney disease was defined as an estimated glomerular filtration rate of <60 mL/min/1.73 m^2^, calculated using the Modification of Diet in Renal Disease equation modified with a Japanese coefficient using the follow-up serum creatinine level [18].

#### Measurements

We assessed the anthropometric parameters of patients and exercise tolerance at the beginning and end of this study. Briefly, anthropometric parameters, including the percentage of body fat, lean body weight, and muscle mass, were measured using bioelectrical impedance analysis (MC-780A; Tanita). The bioelectrical impedance analysis measurements were conducted 2‐3 hours after a meal and before CPET. Exercise capacity was assessed using CPET on a cycle ergometer (Strength Ergo 8, Mitsubishi Electric) with an expiratory gas analysis machine (AE-310S, Minato Medical Science Co, Ltd). After 4 minutes of rest in the sitting position, the patient warmed up for several minutes at 20 W, followed by ramp loading (10 W/min) until they felt exhausted or experienced progressive angina, ST-segment depression (≥2 mm), or sustained tachyarrhythmia [19]. A standard 12-lead electrocardiogram was continuously recorded, and heart rate and BP were recorded every minute during the exercise test. A satisfactory end point of CPET was a respiratory exchange ratio greater than 1.10 [20]. Peak oxygen consumption (peak VO_2_) was defined as the highest oxygen consumption (VO_2_) value recorded during CPET, and the AT point was determined by the V-slope method [21].

Anxiety levels were assessed using a self-administered State Trait Anxiety Inventory (STAI) form at baseline and the end of this study. This inventory consisted of 40 statements about the feelings of the participants and was divided into 2 parts. In part I (comprising 20 statements), patients were instructed to rate the intensity of their current feelings of anxiety (indicating state anxiety) on a scale ranging from 1 (absolutely not) to 4 (very much). In part II (the remaining 20 statements), patients reported the frequency of their general symptoms of anxiety (indicating trait anxiety) on a scale ranging from 1 (hardly ever) to 4 (often). The total score for each part ranges from 20 to 80, with higher scores indicating higher levels of anxiety. The Japanese version of the STAI was used in this study. Health-related quality of life (HR-QOL) was assessed using the Japanese version of the 36-item short form health survey (SF-36) at baseline and at the end of our study [22,23]. SF-36 measures 8 health domains: physical function, physical role, body pain, general health, vitality, social function, emotional role, and mental health. Each domain was scored separately from 0 (indicating the lowest level of functioning) to 100 (the highest level). Activity level was categorized as sedentary (<1.5 METs), lightly active (1.5‐3 METs), moderately active (3‐6 METs), or highly active (>6 METs or ≥145 steps/min sustained for at least 10 min).

#### Recoval System

The Recoval system provides three specific functionalities: (1) visualization of activity level and vital signs during use, (2) personalized activity goal setting, and (3) message-based intervention. The system enables medical personnel to remotely monitor patients’ activity and vital data, including heart rate, steps, and calories burned, through data acquired from supported wearable devices. This data can be visualized on both the medical personnel’s and the patient’s devices, allowing for continuous tracking and analysis. Medical personnel can set individualized exercise goals for each patient, such as target exercise duration, target days per week, target heart rate during exercise, maximum allowable heart rate, and target step count per day. Patients can view these personalized goals and track their progress. Furthermore, the system provides a weekly summary that shows the number of target days versus actual days achieved, as well as comparison of actual step counts to daily targets. The system also facilitates asynchronous communication between patients and medical personnel via a dedicated message screen, where medical personnel can provide tailored feedback, guidance, and motivational messages, while patients can report their conditions and concerns in real time. This communication fosters continuous interaction, enhancing patient engagement and adherence (Multimedia Appendix 1).

#### End Points

The primary outcomes for this study included changes in peak VO_2_ and AT VO_2_ at 12 weeks. The secondary outcomes for this study were changes in CPET parameters, daily activity, anxiety level, and HR-QOL.

### Statistical Analysis

The sample size calculation was based on anticipated changes in peak VO_2_ between the OLC and CON groups, as reportedly previously by comparable studies. We assumed a mean improvement in peak VO_2_ of 2.0 mL/kg/min in the OLC group and 1.0 mL/kg/min in the CON group, estimating this as approximately 80% of the peak VO_2_ increase observed in comparable studies [24]. To determine the sample size, we used a pooled SD of 1.1 mL/kg/min, a 2-sided significance level of 5%, and a power of 80%, which led to an initial requirement of 22 participants per group. To account for an anticipated 10% dropout rate, we adjusted the final sample size to 25 participants per group.

Categorical data are presented as numbers and percentages and were compared using the chi-square test. Continuous variables are expressed as mean (SD) or median (IQR). Between-group comparisons were performed using Student *t* test, while within-group comparisons were performed using a paired *t* test. A *P* value <.05 was considered statistically significant. Effect sizes for the group×time interaction were reported using Cohen *f*, interpreted as small (*f*≥0.10), medium (*f*≥0.25), and large (*f*≥0.40). Missing values were not imputed, and multiplicity was not considered. In this pilot trial, the outcome assessors were blinded to group allocations. All statistical analyses were performed using R (version 4.4.2; R Foundation).

## Results

### Baseline Patient Clinical Characteristics and Blood Test Results

**Figure 2.**
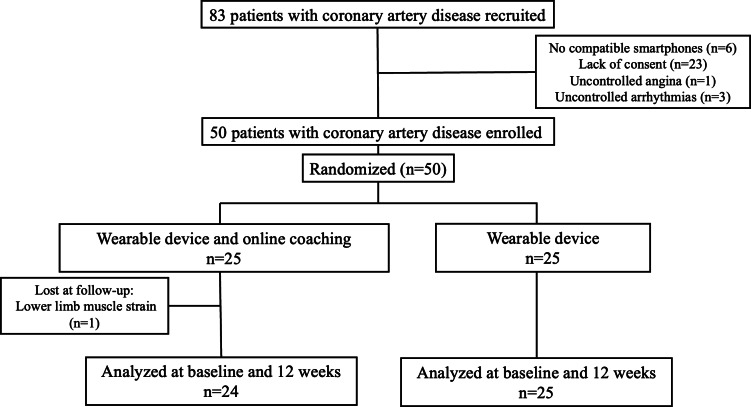
Study flowchart. A total of 83 patients with CAD were initially recruited for this study. Of these, 33 patients were excluded due to various reasons, such as not having compatible smartphones (n=6), lack of consent (n=23), uncontrolled angina (n=1), and uncontrolled arrhythmias (n=3). Finally, 50 patients with CAD were enrolled and classified into 2 groups: the wearable device and online coaching group and the wearable device group. Randomization yielded 25 participants per group. One patient in the wearable device and online coaching group dropped out during the follow-up period. We finally analyzed baseline and 12-week data from a total of 49 patients.

A total of 83 patients with CAD were enrolled in this study. Of these, 6 patients were excluded because they did not have compatible smartphones, 23 were excluded due to lack of consent, 1 was excluded due to uncontrolled angina, and 3 patients were excluded due to uncontrolled arrhythmias. As a result, 50 patients with CAD were finally analyzed and classified into 2 groups, OLC and CON, with randomization yielding 25 participants per group. One patient in the OLC group dropped out during the follow-up period because of lower limb muscle strain. No major adverse cardiac events occurred during this study’s period; these were defined as a composite of cardiovascular death, nonfatal myocardial infarction, nonfatal stroke, and admission for heart failure. Finally, baseline data and data at 12 weeks following intervention from a total of 49 patients were included in the analysis (Figure 2). Table 1 presents the patient backgrounds. The mean age (63.8, SD 6.4 vs 62.6, SD 7.9 years) and sex distribution (n=23, 95.8% vs n=22, 92% male) between the OLC and CON groups were similar. No significant differences were observed between the 2 groups regarding baseline characteristics and coronary risk factors (all *P*>.05). However, the use of β-blockers was significantly higher in the OLC group (n=19, 76%) than in the CON group (n=11, 45.8%; *P*=.03). There were no significant differences in baseline blood data between the 2 groups.

**Table 1.** Baseline clinical characteristics of patients.

		Wearable device and online coaching (n=24)	Wearable device (n=25)	*P* value
Baseline characteristic				
	Age (years), mean (SD)	63.8 (6.4)	62.6 (7.9)	.57
	Sex (male), n (%)	23 (95.8)	22 (92)	.57
	BMI (kg/m^2^), mean (SD)	26.2 (3.8)	25.6 (5.2)	.59
	Appendicular skeletal muscle mass (kg), mean (SD)	25 (4.3)	24.2 (4.1)	.51
	Body fat percentage (%), mean (SD)	23.7 (5.3)	23.1 (11.4)	.81
	Lean body mass (kg), mean (SD)	55.7 (7.5)	53.4 (6.7)	.26
	Acute coronary syndrome, n (%)	12 (50)	13 (52)	.57
	Post PCI^a^, n (%)	21 (87.5)	21 (84)	.73
	Post CABG^b^, n (%)	3 (12.5)	4 (16)	.23
	Hypertension, n (%)	21 (87.5)	3 (12.5)	.60
	Dyslipidemia, n (%)	23 (95.8)	22 (88)	.31
	Diabetes, n (%)	7 (29.2)	12 (48)	.17
	Chronic kidney disease, n (%)	13 (54.2)	18 (72)	.19
Medication				
	Aspirin, n (%)	16 (66.7)	17 (68)	.69
	P2Y12 receptor antagonist, n (%)	11 (45.8)	14 (56)	.48
	β-blocker, n (%)	11 (45.8)	19 (76)	.03
	Calcium channel blocker, n (%)	8 (33.3)	13 (52)	.10
	ACE-I/ARB^c^^d^, n (%)	10 (41.7)	13 (52)	.31
	Statin, n (%)	22 (91.7)	19 (76)	.29
Baseline blood data				
	HbA_1c_^e^ (%), median (IQR)	6.7 (5.9‐6.9)	6.2 (5.9‐6.9)	.51
	Glucose (mg/dL), median (IQR)	100 (92‐126)	106 (93‐130)	.83
	Triglycerides (mg/dL), median (IQR)	118 (91‐148)	98 (76‐148)	.85
	HDL-C^f^ (mg/dL), median (IQR)	46 (42‐51)	51 (44‐60)	.43
	LDL-C^g^ (mg/dL), median (IQR)	69 (60‐79)	62 (58‐69)	.16
	NT-proBNP^h^ (pg/mL), median (IQR)	57 (32‐102)	131 (44‐258)	.12
	High-sensitivity C-reactive protein (mg/mL), median (IQR)	0.05 (0.02‐0.07)	0.07 (0.03‐0.17)	.09
	Creatinine (mg/mL), median (IQR)	0.96 (0.86‐1.03)	0.86 (0.76‐0.91)	.87
	eGFR^i^ (mL/min/1.73m^2^), median (IQR)	61 (58‐70)	70 (58‐80)	.19

a PCI: percutaneous coronary intervention.

b CABG: coronary artery bypass grafting.

c ACE-I: angiotensin-converting enzyme inhibitors.

d ARB: angiotensin receptor blockers.

e HbA_1c_: hemoglobin A_1c_.

f HDL-C: high-density lipoprotein cholesterol.

g LDL-C: low-density lipoprotein cholesterol.

h NT-proBNP: N-terminal probrain natriuretic peptide.

i eGFR: estimated glomerular filtration rate.

### Baseline CPET Parameters, Mental Status, and HR-QOL

A baseline comparison showed no significant differences between the 2 groups regarding CPET parameters, including peak VO_2_ (21.2, SD 3.5 vs 20, SD 5.9; *P*=.39) and AT VO_2_ (13.1, SD 2.3 vs 12.4, SD 3.9; *P*=.40). Similarly, no significant between-group differences were observed concerning mental status measured by STAI and in HR-QOL measured by SF-36 (Table S1 in Multimedia Appendix 2).

### Postintervention Patients’ Clinical Characteristics and Blood Data

After 12 weeks of intervention, both groups exhibited changes in their health metrics (Table 2). In the OLC group, there was a slight reduction in BMI (*P*=.003). Both groups exhibited a decrease in appendicular skeletal muscle mass and a slight increase in body fat percentage; however, these changes were not significant. In terms of blood data, high-density lipoprotein cholesterol levels significantly increased in both groups (*P*=.04).

**Table 2.** Comparison of patient clinical characteristics and blood data at baseline and after 12 weeks in wearable device and online coaching group and wearable device group.

	Wearable device and online coaching				Wearable device				Between-group changes *P* value	Cohen *f*
	Baseline	After 12 weeks	Within-group changes	Within-group *P* value	Baseline	After 12 weeks	Within-group changes	Within-group *P* value		
Patient characteristic										
BMI (kg/m^2^), mean (SD)	26.2 (3.8)	25.9 (3.8)	–0.2 (–0.5 to 0)^a^	.003	25.6 (5.2)	25.5 (5.2)	–0.2 (–0.43 to –0.3)^a^	.003	.16	0.01
Appendicular skeletal muscle mass (kg), mean (SD)	25 (4.3)	24.6 (4.1)	–0.3 (0.2 to –1.1)^a^	.06	24.2 (4.1)	23.8 (3.8)	–0.2 (–1 to 0)^a^	.11	.87	0.004
Body fat percentage (%), mean (SD)	23.7 (5.3)	23.5 (5.7)	0.3 (–1.6 to 1.2)^a^	.58	23.1 (11.4)	23.7 (11.7)	0.7 (–1 to 2.3)^a^	.18	.17	0.02
Blood data										
HbA_1c_^b^ (%), median (IQR)	6.7 (5.9 to 6.9)	6.4 (6.1 to 6.9)	0 (–0.1 to 0)	.32	6.2 (5.9 to 6.9)	6.2 (5.9 to 6.8)	0 (–0.1 to 0.1)	.56	.29	0.05
Glucose (mg/dL), median (IQR)	100 (92 to 126)	111 (100 to 130)	7 (1 to 15)	.11	106 (93 to 130)	105 (96 to 132)	0 (–9 to 15)	.59	.27	0.05
Triglycerides (mg/dL), median (IQR)	118 (91 to 148)	103 (90 to 127)	–5 (–41 to 14)	.29	98 (76 to 148)	110 (80 to 152)	0 (–14 to 4)	.66	.67	0.03
HDL-C^c^ (mg/dL), median (IQR)	46 (42 to 51)	50 (45 to 56)	2 (0 to 3)	.04	51 (44 to 60)	53 (45 to 60)	2 (0 to 7)	.04	.91	0.004
LDL-C^d^ (mg/dL), median (IQR)	69 (60 to 79)	65 (60 to 78)	–2 (–10 to 6)	.35	62 (58 to 69)	65 (55 to 78)	0 (–2 to 10)	.17	.11	0.07
NT-proBNP^e^ (pg/mL), median (IQR)	57 (32 to 102)	58 (25 to 86)	–1 (–39 to 11)	.46	131 (44 to 258)	71 (33 to 195)	–12 (–28 to 0)	.23	.23	0.05
High-sensitivity C-reactive protein (mg/mL), median (IQR)	0.05 (0.02‐0.07)	0.06 (0.03‐0.10)	0 (0 to 0.05)	.81	0.07 (0.03 to 0.17)	0.08 (0.04 to 0.14)	0 (–0.03 to 0.03)	.63	.71	0.04
Creatinine (mg/mL), median (IQR)	0.96 (0.86‐1.03)	0.95 (0.90‐1.04)	0 (–0.1 to 0.06)	.45	0.86 (0.76 to 0.91)	0.94 (0.80 to 1.01)	0.1 (0 to 0.11)	.30	.97	0.001
eGFR^f^ (mL/min/1.73m^2^), median (IQR)	61 (58‐70)	63 (56‐68)	0 (–3 to 7)	.33	70 (58 to 80)	62 (57 to 80)	2 (–2 to 8)	.08	.58	0.02

a Median (IQR).

b HbA_1c_: hemoglobin A_1c_.

c HDL-C: high-density lipoprotein cholesterol.

d LDL-C: low-density lipoprotein cholesterol.

e NT-proBNP: N-terminal probrain natriuretic peptide.

f eGFR: estimated glomerular filtration rate

### Postintervention CPET Parameters, Mental Status, and HR-QOL

Over a period of 12 weeks, changes in CPET parameters and mental status outcomes were observed in both groups (Table 3). In the OLC group, there was a significant increase in peak VO_2_ with a change of 9 (SD 10.3%; *P*<.001), AT VO_2_ with a change of 11.8 (SD 16%; *P*=.001), peak VO_2_/HR with a median change of 0.5 (range −0.2 to 0.7, *P*=.04), and ΔVO_2_/Δload with a mean range 1 (SD 0.8 range 0.4 to 3, *P*=.001). Similarly, in the CON group, significant improvements were noted in peak VO_2_ with a mean change of 8.1 (SD 14%; *P*=.008), AT VO_2_ with a mean change of 12.9 (SD 19.3%; *P*=.002), and ΔVO_2_/Δload with a mean change of 2.0 (range 0.1 to 3.2, *P*=.001). The changes in peak VO_2_, AT VO_2_, peak VO_2_/HR, and ΔVO_2_/Δload over 12 weeks did not show a significant difference between the 2 groups. Concerning mental status, the OLC group showed a significant increase in body pain scores. Conversely, the CON group did not show significant changes in body pain levels.

**Table 3.** Comparison of CPET^a^ parameters and mental status outcomes at baseline and after 12 weeks in wearable device and online coaching group and wearable device group.

			Wearable device and online coaching				Wearable device				Between-group changes *P* value	Cohen *f*
			Baseline, mean (SD)	After 12 weeks, mean (SD)	Within-group changes, median (IQR)	Within-group *P* value	Baseline, mean (SD)	After 12 weeks, mean (SD)	Within-group changes, median (IQR)	Within-group *P* value		
CPET parameters												
	Peak VO_2_^b^ (mL/kg/min)		21.2 (3.5)	23 (3.9)	9% (10.3%)^c^	<.001	20 (5.9)	21.5 (6.3)	8.1% (14%)^c^	.008	.80	0.02
	AT^d^ VO_2_ (mL/kg/min)		13.1 (2.3)	14.4 (2.1)	11.8% (16%)^c^	.001	12.4 (3.9)	13.8 (3.3)	12.9% (19.3%)^c^	.002	.82	0.006
	Resting HR^e^ (beats/min)		69 (11.4)	74 (12.8)	5 (–0.3 to 9.3)	.002	70.9 (13.9)	69.5 (15)	–2 (–5 to 1)	.25	.001	0.12
	Peak HR (beats/min)		140.3 (16.4)	146.3 (17.2)	5.5 (0 to 11.8)	.03	127.6 (27.4)	132.2 (27.5)	6 (0 to 9)	.07	.06	0.02
	Peak VO_2_/HR		11 (2)	11.4 (2)	0.5 (–0.2 to 0.7)	.04	11 (2.3)	11.4 (2.4)	0.4 (–0.6 to 1.1)	.12	.94	0.002
	Ventilation versus VCO_2_^f^ slope		31.8 (4.5)	32.9 (3.9)	1.8 (–0.4 to 3)	.16	31.1 (4.5)	31.8 (5)	0.3 (–0.4 to 1.5)	.22	.70	0.02
	Minimum ventilation/VCO_2_		33.1 (4.4)	33.7 (4.5)	0.5 (0 to 1.2)	.09	33.1 (3.9)	33.3 (4)	0.1 (–0.8 to 1.3)	.75	.43	0.03
	ΔVO_2_/Δload (mL/min/W)		9.2 (1.4)	9.2 (1.4)	1 (0.4 to 3)	.001	8.2 (2)	9.8 (1.6)	2 (0.1 to 3.2)	.001	.53	0.05
	ΔHR/Δload ×100 (beats/W)		60.1 (15)	61.7 (17.3)	1.2 (–5.8 to 6)	.57	52.4 (19.2)	56.3 (20.6)	5.7 (–2.6 to 11)	.09	.38	0.03
Mental status												
	STAI^g^											
		State anxiety	32.4 (7.1)	35.7 (7.8)	4 (0 to 10)	.05	35.1 (8.2)	36 (8.3)	2 (–4 to 6)	.61	.29	0.05
		Trait anxiety	38.8 (10.1)	38.5 (9.3)	1 (–4.3 to 3)	.82	38 (8.5)	39.3 (11.5)	0 (–1 to 6)	.30	.78	0.03
HR-QOL^h^												
	SF-36^i^											
		Physical function	87.3 (12.9)	89.8 (9)	0 (0 to 5)	.26	83.2 (18.3)	85 (19)	0 (0 to 5)	.37	.81	0.01
		Physical role	85.4 (15.5)	88 (15.7)	0 (–12.5 to 18.7)	.46	84.3 (22.5)	84.5 (19.7)	0 (–12.5 to 6.3)	.95	.68	0.03
		Body pain	77.7 (21.7)	67.7 (21.1)	–1 (–21.3 to 0)	.02	66 (26.7)	71.6 (24.2)	0 (–11 to 16)	.33	.03	0.17
		General health	56.5 (21.6)	57.5 (17.5)	0 (–5.8 to 10.5)	.73	54.7 (15.8)	53.8 (19.2)	0 (–5 to 5)	.76	.64	0.03
		Vitality	65.1 (15)	68.2 (14.3)	0 (–1.6 to 12.5)	.30	59.3 (12.2)	62.5 (16.6)	6.3 (–6.2 to 12.5)	.22	.97	0.002
		Social function	95.8 (8.8)	95.3 (8.1)	0 (0 to 0)	.77	89.5 (12.8)	90 (16.9)	0 (0 to 12.5)	.89	.80	0.02
		Emotional role	88.2 (14.3)	93.8 (10.8)	0 (0 to 25)	.13	79.3 (26.7)	87.3 (17.9)	0 (–8.3 to 8.3)	.10	.68	0.03
		Mental health	79.6 (11.9)	78.1 (15.6)	0 (–5 to 5)	.65	74 (14.1)	75.2 (15.9)	0 (–5 to 5)	.64	.51	0.05

a CPET: cardiopulmonary exercise test.

b VO2: oxygen consumption.

c Mean (SD).

d AT: anaerobic threshold.

e HR: heart rate.

f VCO_2_: carbon dioxide production.

g STAI: State-Trait Anxiety Inventory.

h HR-QOL: health-related quality of life.

i SF-36: 36-Item short form health survey.

### Daily Activity

**Figure 3.**
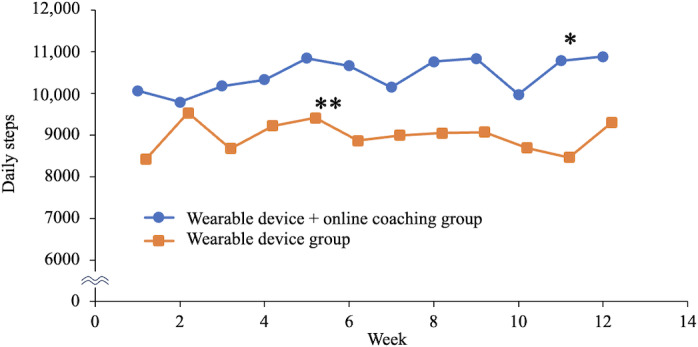
The average daily steps taken each week. * *P*<.05 Student *t* test (between-groups), ** *P*<.05 paired *t* test (within-group).

**Figure 4.**
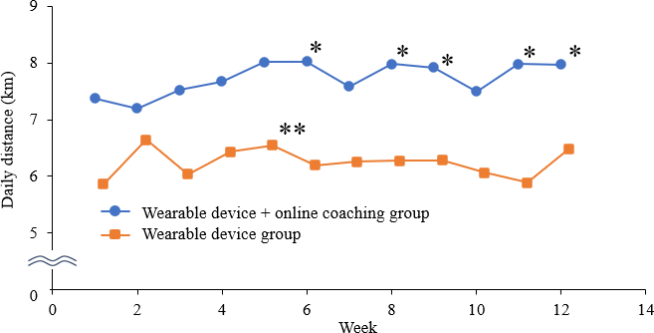
The average daily distance taken each week. * *P*<.05 Student *t* test (between-groups), ** *P*<.05 paired *t* test (within-group).

**Figure 5.**
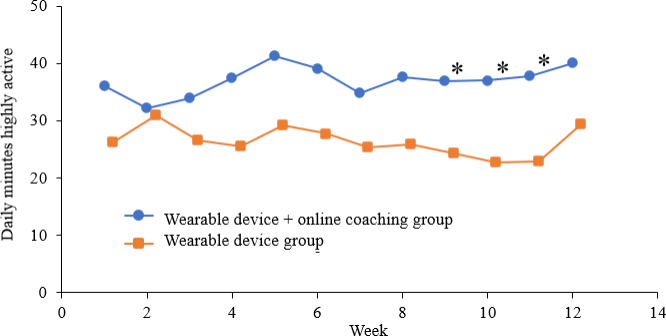
The average daily minutes of highly active taken each week. * *P*<.05 Student *t* test (between-groups).

**Figure 6.**
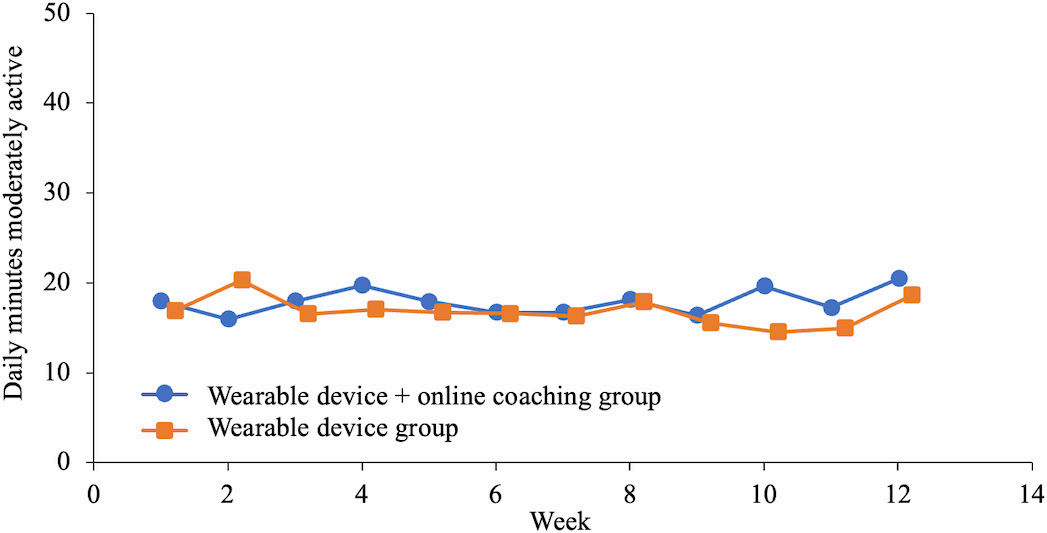
The average daily minutes of moderately active taken each week.

The parameters measured by the wearable device are shown in Figures 3-6. Figure 3 displays the average number of daily steps taken each week. Although the increase in steps in the OLC group was not significant, a notable increase in the number of steps was observed. After 11 weeks, there was a significant increase in the number of steps compared with that in the CON group (10,785 steps vs 8463 steps; *P*=.03)*.* Figure 4 displays the average daily travel distance per week. The distance covered was significantly longer for OLC group, especially in the latter period, compared with that in the CON group (8 km vs 6.5 km; *P*=.003). Of activity level, the daily duration of high activity was significantly longer for the OLC group after 9 weeks than that for the CON group (37 min vs 21.7 min; *P*=.03). However, there was no significant difference in the duration of moderate activity between the 2 groups (Figures5  6).

## Discussion

### Principal Results

We investigated the effects of remote CR using wearable devices with and without OLC in patients with CAD. The major findings of this study were as follows: (1) using wearable devices and providing a home exercise program based on CPET for 3 months significantly improved peak VO_2_ and AT VO_2_; (2) the OLC group demonstrated significantly higher physical activity (daily distance and daily duration of high activity) in the latter half of the intervention than did the CON group; and (3) during the research period, there were no dropouts owing to the use of wearable device or OLC.

### Limitations

This study had some limitations. First, there was a potential selection bias in the cases enrolled in this study, as it may have included many individuals with high health consciousness and tolerance for wearable devices and desire for OLC. These factors may limit the generalizability of our findings to population groups with lower levels of health consciousness or technological proficiency. Second, this was a single-center study conducted primarily among Japanese patients, with a small sample size and a short follow-up period of 12 weeks. Regional differences and cultural factors may have influenced the results. Furthermore, the predominance of the male sex in this study’s cohort may also limit the applicability of the findings to females, older adults, or different ethnic groups. Additionally, varying levels of digital literacy and access to technology could impact the effectiveness of remote CR programs, both within Japan and internationally. Third, the power analysis indicates that this study is sufficiently powered to detect large effect size differences between the OLC group and CON group, specifically a Cohen *d* of 0.8 with a sample size of 50. These effect sizes are rather large and may be unrealistic for the intervention being studied, suggesting that this study is underpowered for detecting more moderate or smaller effect sizes. This study serves primarily as a proof of concept. Future interventional studies with larger sample sizes, longer follow-up durations, and more diverse populations are needed to confirm the findings and evaluate long-term adherence and sustained benefits of such programs.

### Comparison With Prior Work

Peak VO_2_, measured using the CPET, represents the maximal ability of the body to transport and use oxygen. It is widely recognized as a definitive measure of exercise capacity [25-28]. In patients with CAD, peak VO_2_ is associated with all-cause mortality and cardiovascular events [29-32]. AT VO_2_, similar to peak VO_2_, is considered a prognostic indicator [33,34]. It is recommended to prescribe exercise below the AT to establish a safe exercise tolerance range of intensity for patients [5]. In this study, by providing a home exercise program based on AT VO_2_ and using wearable devices, we identified significant enhancements in both peak VO_2_ and AT VO_2_ over a 12-week intervention period, irrespective of the presence or absence of OLC. Additionally, we observed a significant improvement in ΔVO_2_/Δload. This parameter serves as an indicator of oxygen transport to the peripheral muscles, reflecting the degree of increase in cardiac output during exercise [35]. It has been reported that ΔVO_2_/Δload is an independent prognostic factor in patients with cardiovascular disease [36]. In previous studies, it has been reported that the use of physical activity trackers for more than 12 weeks improves peak VO_2_ in patients with CAD, however there is no mention of CPET parameters other than peak VO_2_, such as AT VO_2_ or ΔVO_2_/Δload [15,37]. Additionally, the patient group had already participated in a phase II CR program. Our study targeted patients in phase III and did not consider whether they had participated in the CR program, suggesting that a combined approach using wearable devices and a CPET-based home exercise regimen offers a promising strategy for remote CR in phase III patients with CAD, potentially leading to improved prognosis and enhanced exercise capacity. However, this study was a short-term intervention of 12 weeks, and determining its effects requires a long-term intervention and follow-up.

The World Health Organization guidelines recommend at least 150‐300 minutes of moderate physical activity weekly, at least 75‐150 minutes of vigorous physical activity weekly, or an equivalent combination of both [38]. It has been reported that moderate-to-vigorous-intensity activity and vigorous physical activity, as measured by wearable devices, have a significant negative correlation with the risk of all-cause mortality, cardiovascular death, and onset of heart failure [8,9,39]. In this study, the OLC group had a significantly increased daily distance travelled compared with that by the CON group in the latter period. Additionally, while there was no significant difference in the time spent moderately active (3‐6 METs), the time spent highly active (>6 METs) significantly increased in the OLC group during the latter half of the intervention. To date, no study has examined the significance of OLC using wearable devices. In this study, weekly OLC had a positive effect on daily activity, particularly vigorous activity, in the latter half of the remote CR program. One possible reason is that OLC may help maintain the motivation to continue exercise habits and provide guidance on appropriate exercise methods. Notably, 1 participant in the OLC group dropped out owing to calf muscle strain, and the OLC group experienced increased body pain as measured by HR-QOL. This suggests that an increase in activity may lead to musculoskeletal problems. To safely continue remote-CR, it is important to not only increase vigorous activity but also to enhance moderate activity. Patient education and OLC are crucial in this regard.

With the advancement of digital devices, including wearable devices and smartphones, it is anticipated that remote-CR will continue to evolve in the future [40]. Prior research on remote CR using these digital platforms has primarily focused on relatively healthy patients in stable condition. Nonetheless, reported dropout rates in studies with a duration under 12 weeks ranged 3%‐13%, even among patients who demonstrated a degree of digital device usage tolerance [41-43]. In this study, the patients were predominantly male (>90%), with an average age of 63.1 (SD 7.2) years, indicating that the cohort was less likely to be affected by the digital divide. Future research must prioritize the validation of remote CR in older and more vulnerable populations, while also assessing the efficacy and safety of interventions over extended periods.

### Conclusions

Remote CR using wearable devices and a real-time monitoring system significantly improved exercise capacity in patients with CAD over a 12-week intervention period. Physical activity improved significantly with regular OLC. Future research should focus on diverse patient populations and longer intervention duration to validate these findings.

## Supplementary material

10.2196/63797 Multimedia Appendix 1 Overview of the Recoval system. This supplemental material provides an overview of the Recoval system, a platform designed to enable medical personnel to remotely monitor and manage patients' activity and vital data through wearable devices. By integrating patient data from smart devices, the system facilitates remote interventions and personalized care.

10.2196/63797 Multimedia Appendix 2 Baseline comparison of CPET parameters and mental status. CPET: cardiopulmonary exercise test.

10.2196/63797 Checklist 1 CONSORT-EHEALTH checklist.

## Abbreviations

AT: anaerobic threshold
BP: blood pressure
CAD: coronary artery disease
CON: control wearable device
CONSORT: Consolidated Standards of Reporting Trials
CPET: cardiopulmonary exercise test
CR: cardiac rehabilitation
HR-QOL: health-related quality of life
MET: metabolic-equivalent task
OLC: online coaching
SF-36: 36-item short form health survey
STAI: State Trait Anxiety Inventory
VO_2_: oxygen consumption
