# Supplementary figures and images for: Wearable Devices in Remote Cardiac Rehabilitation With and Without Weekly Online Coaching for Patients With Coronary Artery Disease: Randomized Controlled Trial
Source: JMIR Mhealth Uhealth. 2025 May 12;13:e63797. doi: 10.2196/63797 (PMC12088619; doi:10.2196/63797)

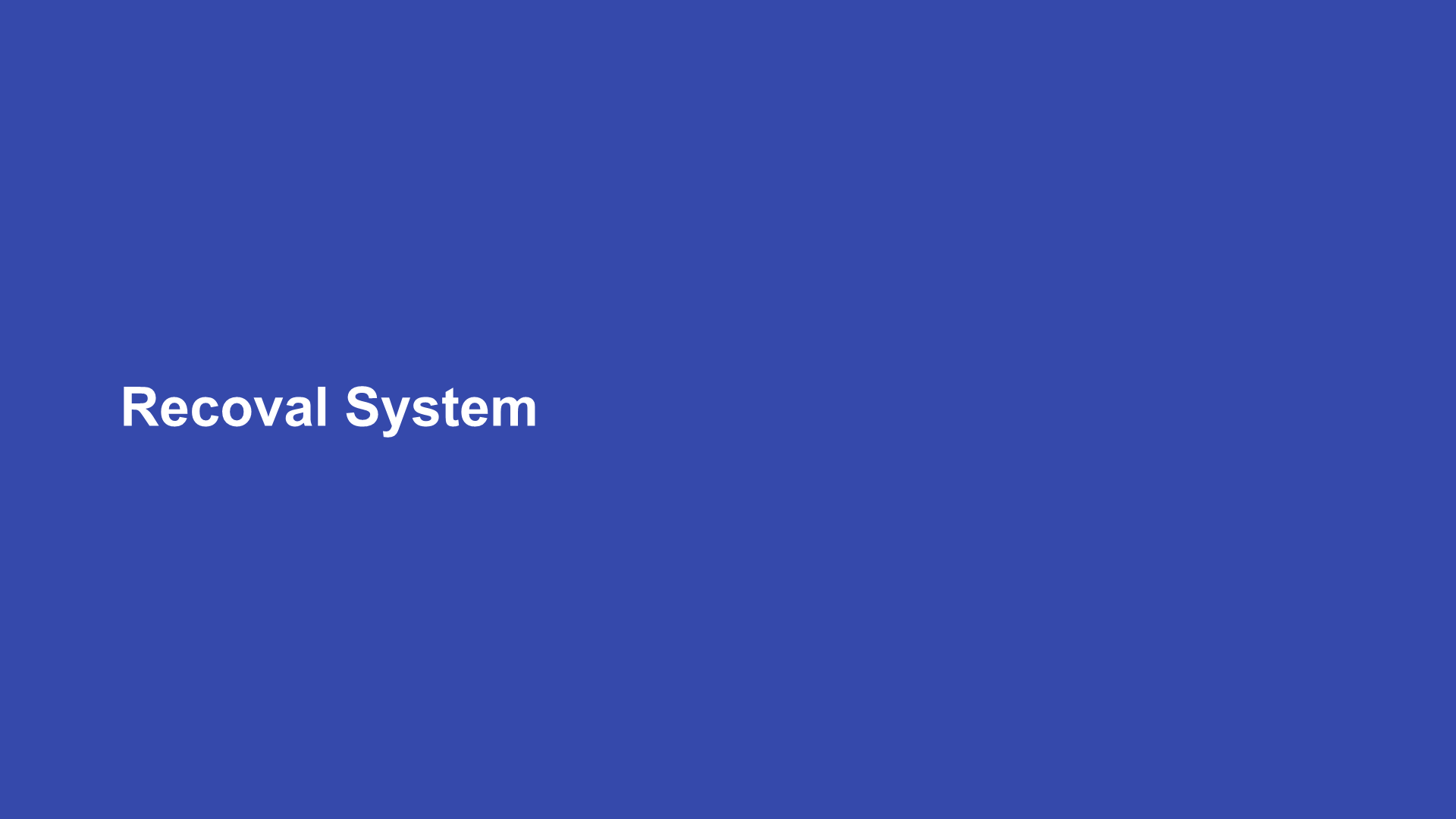

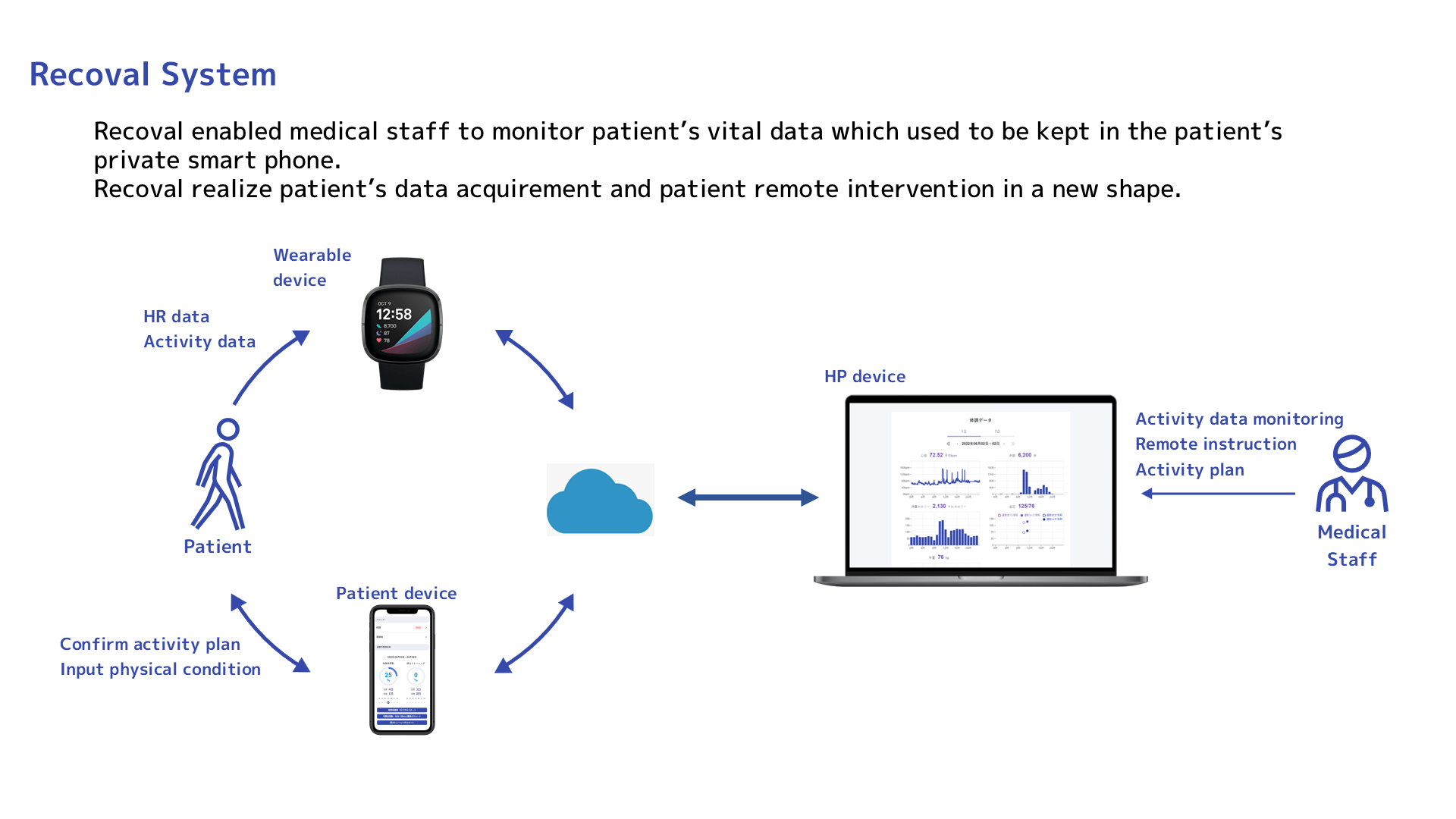

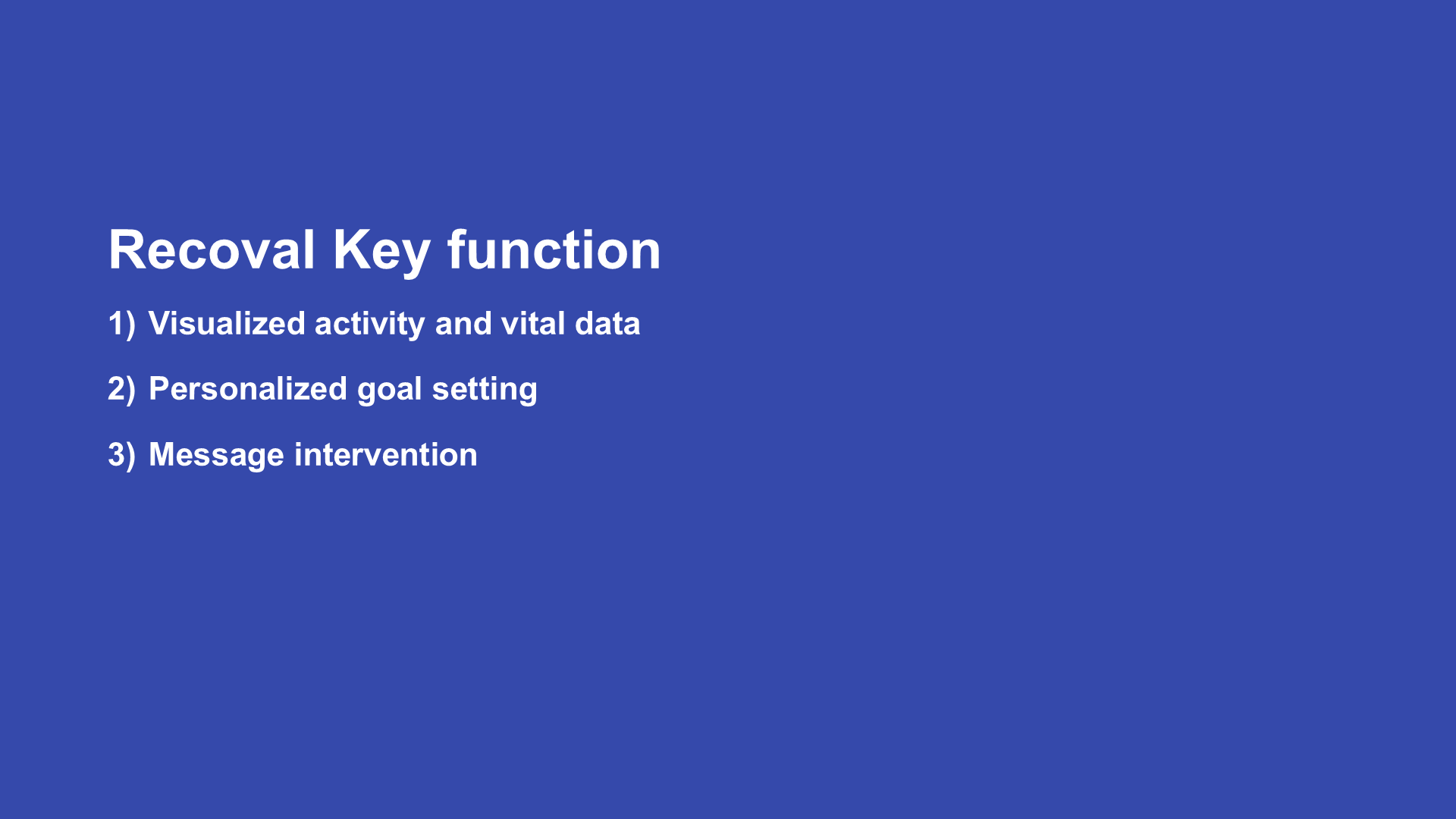

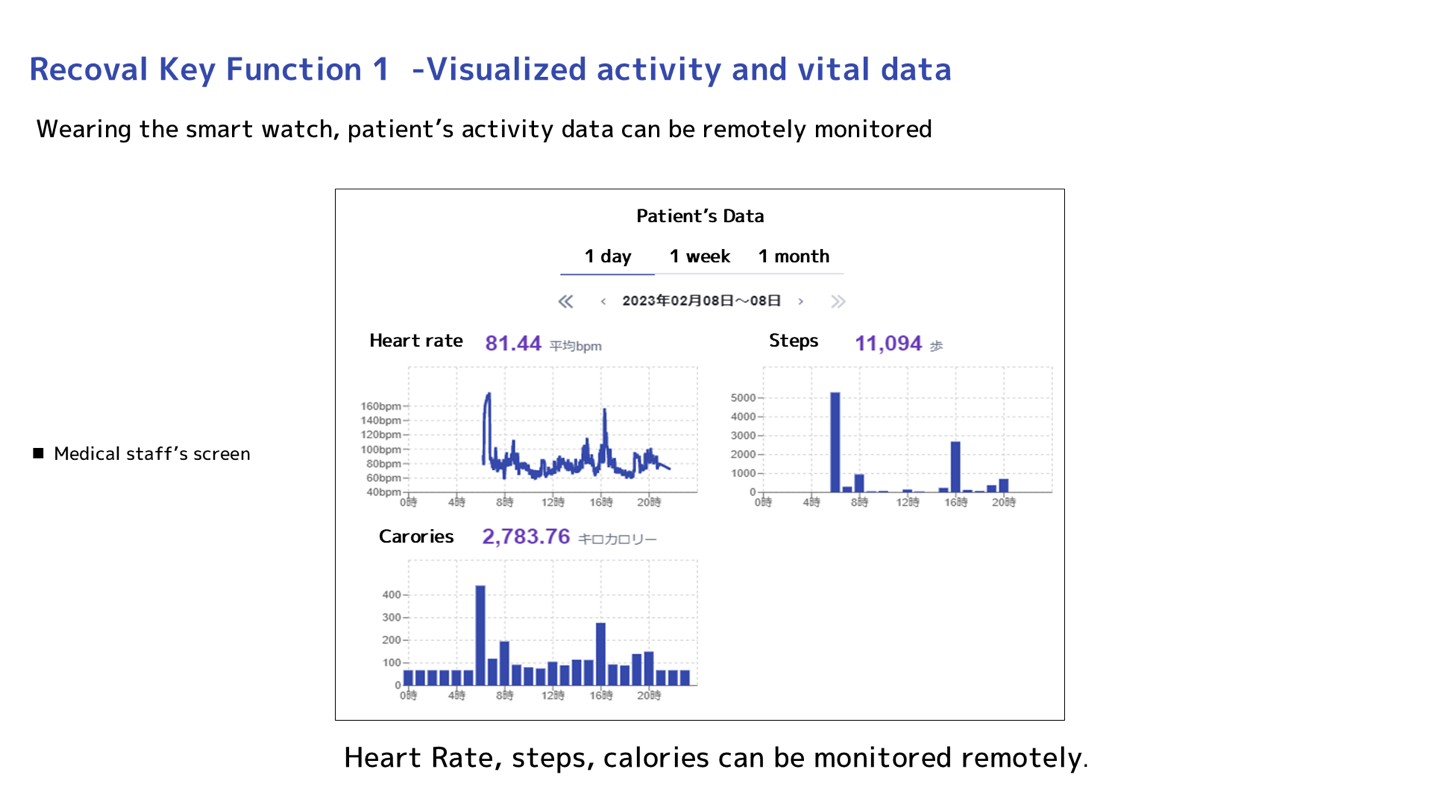

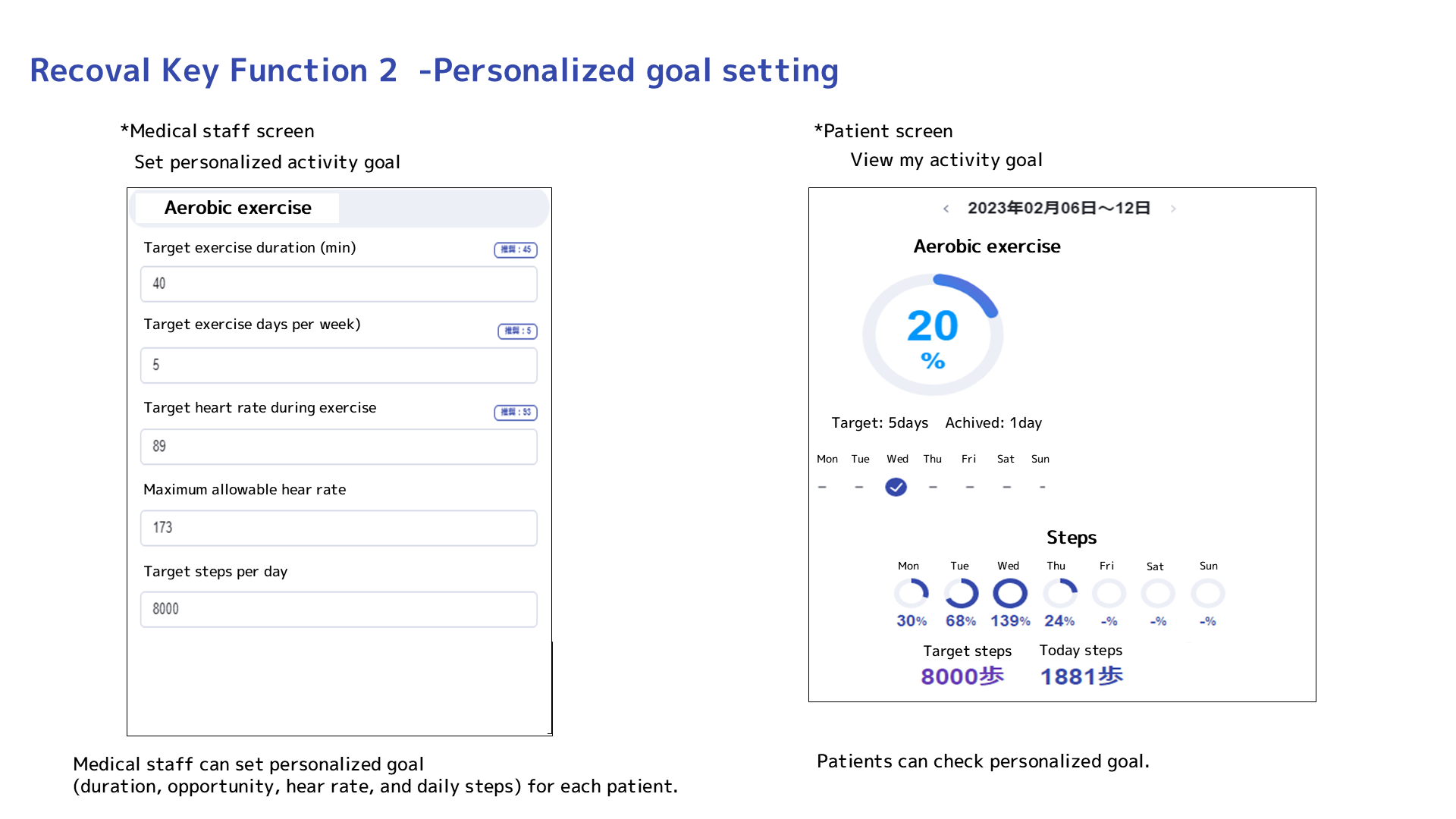

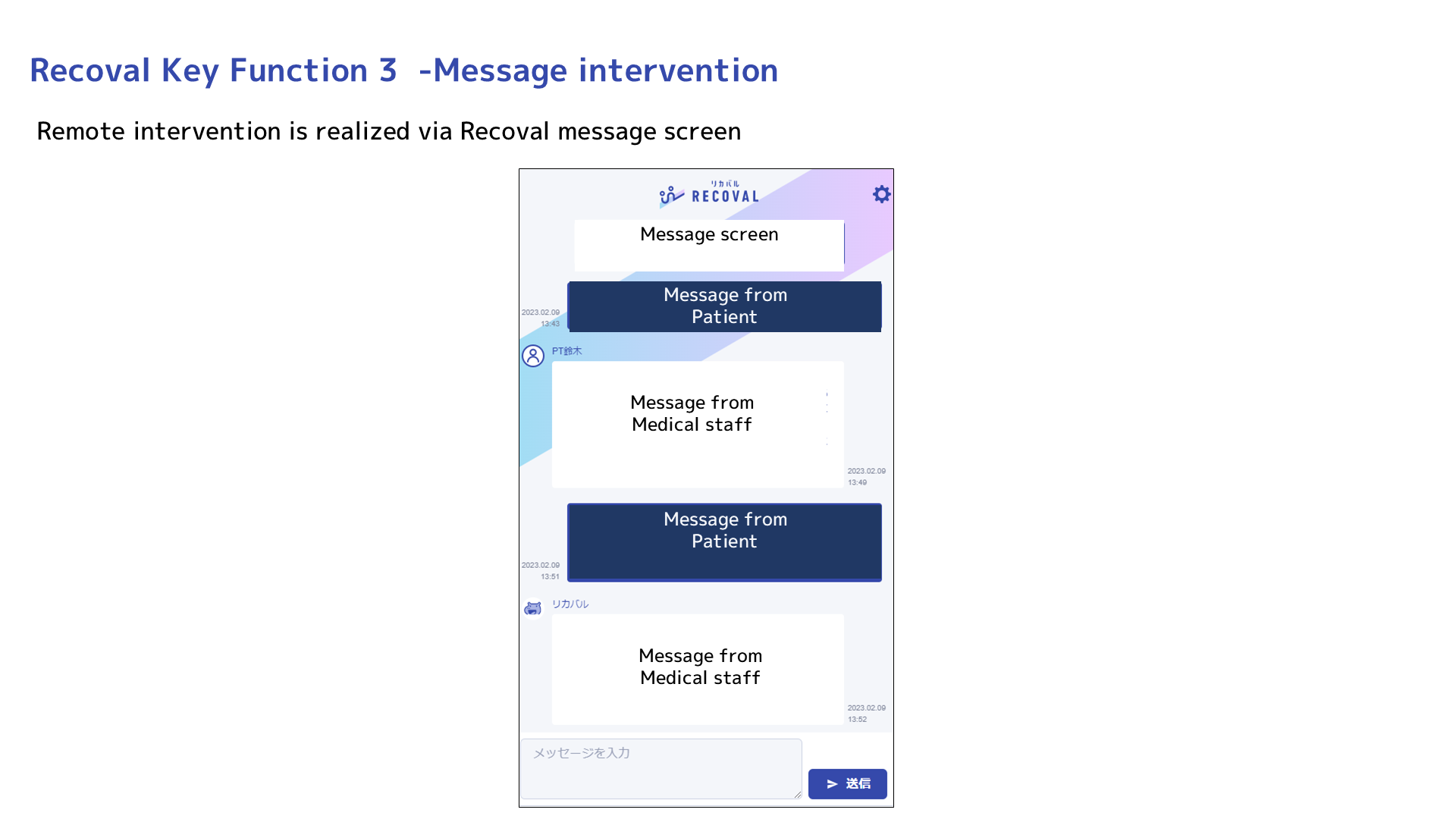

Supplement: Multimedia Appendix 1 [file mhealth-v13-e63797-s001.docx]
