# Supplementary material for: Wearable Devices in Remote Cardiac Rehabilitation With and Without Weekly Online Coaching for Patients With Coronary Artery Disease: Randomized Controlled Trial
Source: JMIR Mhealth Uhealth. 2025 May 12;13:e63797. doi: 10.2196/63797 (PMC12088619; doi:10.2196/63797)
Supplement: Multimedia Appendix 2 [file mhealth-v13-e63797-s002.docx]

**Table S1.** Baseline comparison of CPET parameters and mental status.

|  | **Wearable device**  **+**  **Online coaching** | **Wearable device** |  |
| --- | --- | --- | --- |
|  | **(n = 24)** | **(n = 25)** | ***p*** |
| **CPET parameters** |  |  |  |
| Peak VO_2_ (mL/kg/min) | 21.2 ± 3.5 | 20.0 ± 5.9 | 0.39 |
| AT VO_2_ (mL/kg/min) | 13.1 ± 2.3 | 12.4 ± 3.9 | 0.40 |
| Resting HR (beats/min) | 69.0 ± 11.4 | 70.9 ± 13.9 | 0.60 |
| Peak HR (beats/min) | 140.3 ± 16.4 | 127.6 ± 27.4 | 0.05 |
| Peak VO_2_/HR | 11.0 ± 2.0 | 11.0 ± 2.3 | 0.99 |
| VE vs. VCO_2_ slope | 31.8 ± 4.5 | 31.1 ± 4.5 | 0.59 |
| Minimum VE/VCO_2_ | 33.1 ± 4.4 | 33.1 ± 3.9 | 0.98 |
| ΔVO_2_/ΔLoad (mL/min/W) | 9.2 ± 1.4 | 8.2 ± 2.0 | 0.06 |
| ΔHR/ΔLoad × 100 (beats/W) | 60.1 ± 15.0 | 52.4 ± 19.2 | 0.13 |
| **Mental status** |  |  |  |
| STAI |  |  |  |
| State anxiety | 32.4 ± 7.1 | 35.1 ± 8.2 | 0.21 |
| Trait anxiety | 38.8 ± 10.1 | 38.0 ± 8.5 | 0.78 |
| **HR-QOL** |  |  |  |
| SF-36 |  |  |  |
| Physical function | 87.3 ± 12.9 | 83.2 ± 18.3 | 0.37 |
| Physical role | 85.4 ± 15.5 | 84.3 ± 22.5 | 0.83 |
| Body pain | 77.7 ± 21.7 | 66.0 ± 26.7 | 0.10 |
| General health | 56.5 ± 21.6 | 54.7 ± 15.8 | 0.73 |
| Vitality | 65.1 ± 15.0 | 59.3 ± 12.2 | 0.14 |
| Social function | 95.8 ± 8.8 | 89.5 ± 12.8 | 0.05 |
| Emotional role | 88.2 ± 14.3 | 79.3 ± 26.7 | 0.16 |
| Mental health | 79.6 ± 11.9 | 74.0 ± 14.1 | 0.14 |

AT, anaerobic threshold; CPET, cardiopulmonary exercise test; HR, heart rate; HR-QOL; health-related quality of life, SF-36, 36-Item short form health survey; STAI, state-trait anxiety inventory form; VCO2, carbon dioxide production; VE, minute ventilation; VO2, oxygen consumption.
