# Supplementary material for: Wearable Devices in Remote Cardiac Rehabilitation With and Without Weekly Online Coaching for Patients With Coronary Artery Disease: Randomized Controlled Trial
Source: JMIR Mhealth Uhealth. 2025 May 12;13:e63797. doi: 10.2196/63797 (PMC12088619; doi:10.2196/63797)
Supplement: Checklist 1 [file mhealth-v13-e63797-s003.pdf]

CONSORT-EHEALTH (V 1.6.1) - Submission/Publication Form

The CONSORT-EHEALTH checklist is intended for authors of randomized trials evaluating web-based and Internet-based applications/interventions, including mobile interventions, electronic games (incl multiplayer games), social media, certain telehealth applications, and other interactive and/or networked electronic applications. Some of the items (e.g. all subitems under item 5 - description of the intervention) may also be applicable for other study designs.

The goal of the CONSORT EHEALTH checklist and guideline is to be
a) a guide for reporting for authors of RCTs,
b) to form a basis for appraisal of an ehealth trial (in terms of validity)

CONSORT-EHEALTH items/subitems are MANDATORY reporting items for studies published in the Journal of Medical Internet Research and other journals / scientific societies endorsing the checklist.

Items numbered 1., 2., 3., 4a., 4b etc are original CONSORT or CONSORT-NPT (non-pharmacologic treatment) items.
Items with Roman numerals (i., ii, iii, iv etc.) are CONSORT-EHEALTH extensions/clarifications.

As the CONSORT-EHEALTH checklist is still considered in a formative stage, we would ask that you also RATE ON A SCALE OF 1-5 how important/useful you feel each item is FOR THE PURPOSE OF THE CHECKLIST and reporting guideline (optional).

Mandatory reporting items are marked with a red *.
In the textboxes, either copy & paste the relevant sections from your manuscript into this form - please include any quotes from your manuscript in QUOTATION MARKS,
or answer directly by providing additional information not in the manuscript, or elaborating on why the item was not relevant for this study.

YOUR ANSWERS WILL BE PUBLISHED AS A SUPPLEMENTARY FILE TO YOUR PUBLICATION IN JMIR AND ARE CONSIDERED PART OF YOUR PUBLICATION (IF ACCEPTED).
Please fill in these questions diligently. Information will not be copyedited, so please use proper spelling and grammar, use correct capitalization, and avoid abbreviations.

DO NOT FORGET TO SAVE AS PDF _AND_ CLICK THE SUBMIT BUTTON SO YOUR ANSWERS ARE IN OUR DATABASE !!!

Citation Suggestion (if you append the pdf as Appendix we suggest to cite this paper in the caption):
Eysenbach G, CONSORT-EHEALTH Group
CONSORT-EHEALTH: Improving and Standardizing Evaluation Reports of Web-based and Mobile Health Interventions
J Med Internet Res 2011;13(4):e126

URL: http://www.jmir.org/2011/4/e126/
doi: 10.2196/jmir.1923
PMID: 22209829

r-nishio@juntendo.ac.jp アカウントを切り替える

共有なし

下書きを保存しました

* 必須の質問です

Your name *

First Last

Ryota Nishio

Primary Affiliation (short), City, Country *

University of Toronto, Toronto, Canada

Department of Cardiovascular Biology and Me

Your e-mail address *

abc@gmail.com

r-nishio@juntendo.ac.jp

Title of your manuscript *

Provide the (draft) title of your manuscript.

Effects of Remote Cardiac Rehabilitation Using Wearable Devices with and without Weekly Online Coaching in CAD Patients: A Randomized trial

Name of your App/Software/Intervention *

If there is a short and a long/alternate name, write the short name first and add the long name in brackets.

Recoval ©

Evaluated Version (if any)

e.g. "V1", "Release 2017-03-01", "Version 2.0.27913"

回答を入力

Language(s) *

What language is the intervention/app in? If multiple languages are available, separate by comma (e.g. "English, French")

Japanese

URL of your Intervention Website or App

e.g. a direct link to the mobile app on app in appstore (itunes, Google Play), or URL of the website. If the intervention is a DVD or hardware, you can also link to an Amazon page.

https://m3comlp.m3.com/lp/sapplym/recoval.

URL of an image/screenshot (optional)

回答を入力

Accessibility *

Can an enduser access the intervention presently?

- ( ) access is free and open
- (x) access only for special usergroups, not open
- ( ) access is open to everyone, but requires payment/subscription/in-app purchases
- ( ) app/intervention no longer accessible
- ( ) その他:

Primary Medical Indication/Disease/Condition *

e.g. "Stress", "Diabetes", or define the target group in brackets after the condition, e.g. "Autism (Parents of children with)", "Alzheimers (Informal Caregivers of)"

Coronary artery disease

Primary Outcomes measured in trial *

comma-separated list of primary outcomes reported in the trial

Changes in peak VO2 and anaerobic threshold

Secondary/other outcomes

Are there any other outcomes the intervention is expected to affect?

changes in CPET parameters, daily activity, anxiety levels, and health-related quality of life (HR-QOL).

Recommended "Dose" *

What do the instructions for users say on how often the app should be used?

- ○ Approximately Daily
- ◉ Approximately Weekly
- ○ Approximately Monthly
- ○ Approximately Yearly
- ○ "as needed"
- ○ その他:

Approx. Percentage of Users (starters) still using the app as recommended after 3 months *

- ○ unknown / not evaluated
- ○ 0-10%
- ○ 11-20%
- ○ 21-30%
- ○ 31-40%
- ○ 41-50%
- ○ 51-60%
- ○ 61-70%
- ○ 71%-80%
- ○ 81-90%
- ◉ 91-100%
- ○ その他:

Overall, was the app/intervention effective? *

- ○ yes: all primary outcomes were significantly better in intervention group vs control
- ◉ partly: SOME primary outcomes were significantly better in intervention group vs control
- ○ no statistically significant difference between control and intervention
- ○ potentially harmful: control was significantly better than intervention in one or more outcomes
- ○ inconclusive: more research is needed
- ○ その他:

Article Preparation Status/Stage *

At which stage in your article preparation are you currently (at the time you fill in this form)

- ○ not submitted yet - in early draft status
- ○ not submitted yet - in late draft status, just before submission
- ○ submitted to a journal but not reviewed yet
- ○ submitted to a journal and after receiving initial reviewer comments
- ◉ submitted to a journal and accepted, but not published yet
- ○ published
- ○ その他:

Journal *

If you already know where you will submit this paper (or if it is already submitted), please provide the journal name (if it is not JMIR, provide the journal name under "other")

- ○ not submitted yet / unclear where I will submit this
- ○ Journal of Medical Internet Research (JMIR)
- ◉ JMIR mHealth and UHealth
- ○ JMIR Serious Games
- ○ JMIR Mental Health
- ○ JMIR Public Health
- ○ JMIR Formative Research
- ○ Other JMIR sister journal
- ○ その他:

Is this a full powered effectiveness trial or a pilot/feasibility trial? *

- ◉ Pilot/feasibility
- ○ Fully powered

Manuscript tracking number *

If this is a JMIR submission, please provide the manuscript tracking number under "other" (The ms tracking number can be found in the submission acknowledgement email, or when you login as author in JMIR. If the paper is already published in JMIR, then the ms tracking number is the four-digit number at the end of the DOI, to be found at the bottom of each published article in JMIR)

- ○ no ms number (yet) / not (yet) submitted to / published in JMIR
- ◉ その他: JMIR Mhealth Uhealth 63797

TITLE AND ABSTRACT

1a) TITLE: Identification as a randomized trial in the title

1a) Does your paper address CONSORT item 1a? *

I.e does the title contain the phrase "Randomized Controlled Trial"? (if not, explain the reason under "other")

- (●) yes
- ( ) その他:

1a-i) Identify the mode of delivery in the title

Identify the mode of delivery. Preferably use “web-based” and/or “mobile” and/or “electronic game” in the title. Avoid ambiguous terms like “online”, “virtual”, “interactive”. Use “Internet-based” only if Intervention includes non-web-based Internet components (e.g. email), use “computer-based” or “electronic” only if offline products are used. Use “virtual” only in the context of “virtual reality” (3-D worlds). Use “online” only in the context of “online support groups”. Complement or substitute product names with broader terms for the class of products (such as “mobile” or “smart phone” instead of “iphone”), especially if the application runs on different platforms.

	1	2	3	4	5	
subitem not at all important	( )	( )	( )	( )	(●)	essential

選択を解除

Does your paper address subitem 1a-i? *

Copy and paste relevant sections from manuscript title (include quotes in quotation marks "like this" to indicate direct quotes from your manuscript), or elaborate on this item by providing additional information not in the ms, or briefly explain why the item is not applicable/relevant for your study

”Using Wearable Devices with and without Weekly Online Coaching”

1a-ii) Non-web-based components or important co-interventions in title

Mention non-web-based components or important co-interventions in title, if any (e.g., “with telephone support”).

	1	2	3	4	5	
subitem not at all important	○	○	○	○	◉	essential

選択を解除

Does your paper address subitem 1a-ii?

Copy and paste relevant sections from manuscript title (include quotes in quotation marks "like this" to indicate direct quotes from your manuscript), or elaborate on this item by providing additional information not in the ms, or briefly explain why the item is not applicable/relevant for your study

”with and without Weekly Online Coaching””

1a-iii) Primary condition or target group in the title

Mention primary condition or target group in the title, if any (e.g., “for children with Type I Diabetes”) Example: A Web-based and Mobile Intervention with Telephone Support for Children with Type I Diabetes: Randomized Controlled Trial

	1	2	3	4	5	
subitem not at all important	○	○	○	○	◉	essential

選択を解除

Does your paper address subitem 1a-iii? *

Copy and paste relevant sections from manuscript title (include quotes in quotation marks "like this" to indicate direct quotes from your manuscript), or elaborate on this item by providing additional information not in the ms, or briefly explain why the item is not applicable/relevant for your study

”in CAD Patients”

1b) ABSTRACT: Structured summary of trial design, methods, results, and conclusions

NPT extension: Description of experimental treatment, comparator, care providers, centers, and blinding status.

1b-i) Key features/functionalities/components of the intervention and comparator in the METHODS section of the ABSTRACT

Mention key features/functionalities/components of the intervention and comparator in the abstract. If possible, also mention theories and principles used for designing the site. Keep in mind the needs of systematic reviewers and indexers by including important synonyms. (Note: Only report in the abstract what the main paper is reporting. If this information is missing from the main body of text, consider adding it)

	1	2	3	4	5	
subitem not at all important	○	○	○	○	◉	essential

選択を解除

Does your paper address subitem 1b-i? *

Copy and paste relevant sections from the manuscript abstract (include quotes in quotation marks "like this" to indicate direct quotes from your manuscript), or elaborate on this item by providing additional information not in the ms, or briefly explain why the item is not applicable/relevant for your study

""We enrolled 50 patients with CAD in a remote CR program in this randomised, open-label, single-centre pilot trial (phase III)."

1b-ii) Level of human involvement in the METHODS section of the ABSTRACT

Clarify the level of human involvement in the abstract, e.g., use phrases like “fully automated” vs. “therapist/nurse/care provider/physician-assisted” (mention number and expertise of providers involved, if any). (Note: Only report in the abstract what the main paper is reporting. If this information is missing from the main body of text, consider adding it)

	1	2	3	4	5	
subitem not at all important	○	○	○	●	○	essential

選択を解除

Does your paper address subitem 1b-ii?

Copy and paste relevant sections from the manuscript abstract (include quotes in quotation marks "like this" to indicate direct quotes from your manuscript), or elaborate on this item by providing additional information not in the ms, or briefly explain why the item is not applicable/relevant for your study

”all patients were assigned a CPET-based home exercise program and were provided with a wearable device (Fitbit Sense)”

1b-iii) Open vs. closed, web-based (self-assessment) vs. face-to-face assessments in the METHODS section of the ABSTRACT

Mention how participants were recruited (online vs. offline), e.g., from an open access website or from a clinic or a closed online user group (closed usergroup trial), and clarify if this was a purely web-based trial, or there were face-to-face components (as part of the intervention or for assessment). Clearly say if outcomes were self-assessed through questionnaires (as common in web-based trials). Note: In traditional offline trials, an open trial (open-label trial) is a type of clinical trial in which both the researchers and participants know which treatment is being administered. To avoid confusion, use “blinded” or “unblinded” to indicated the level of blinding instead of “open”, as “open” in web-based trials usually refers to “open access” (i.e. participants can self-enrol). (Note: Only report in the abstract what the main paper is reporting. If this information is missing from the main body of text, consider adding it)

	1	2	3	4	5	
subitem not at all important	○	○	○	◉	○	essential

選択を解除

Does your paper address subitem 1b-iii?

Copy and paste relevant sections from the manuscript abstract (include quotes in quotation marks "like this" to indicate direct quotes from your manuscript), or elaborate on this item by providing additional information not in the ms, or briefly explain why the item is not applicable/relevant for your study

”We enrolled 50 patients with CAD in a remote CR program in this randomised, open-label, single-centre pilot trial (phase III)”

1b-iv) RESULTS section in abstract must contain use data

Report number of participants enrolled/assessed in each group, the use/uptake of the intervention (e.g., attrition/adherence metrics, use over time, number of logins etc.), in addition to primary/secondary outcomes. (Note: Only report in the abstract what the main paper is reporting. If this information is missing from the main body of text, consider adding it)

	1	2	3	4	5	
subitem not at all important	○	○	○	◉	○	essential

選択を解除

Does your paper address subitem 1b-iv?

Copy and paste relevant sections from the manuscript abstract (include quotes in quotation marks "like this" to indicate direct quotes from your manuscript), or elaborate on this item by providing additional information not in the ms, or briefly explain why the item is not applicable/relevant for your study

”The patients were randomly assigned to an intervention group with online coaching (OLC; n=25) or a control group (CON; n=25)”
” One patient in the OLC group dropped out due to lower limb muscle strain.”

1b-v) CONCLUSIONS/DISCUSSION in abstract for negative trials

Conclusions/Discussions in abstract for negative trials: Discuss the primary outcome - if the trial is negative (primary outcome not changed), and the intervention was not used, discuss whether negative results are attributable to lack of uptake and discuss reasons. (Note: Only report in the abstract what the main paper is reporting. If this information is missing from the main body of text, consider adding it)

	1	2	3	4	5	
subitem not at all important	○	○	◉	○	○	essential

選択を解除

Does your paper address subitem 1b-v?

Copy and paste relevant sections from the manuscript abstract (include quotes in quotation marks "like this" to indicate direct quotes from your manuscript), or elaborate on this item by providing additional information not in the ms, or briefly explain why the item is not applicable/relevant for your study

Our study was not a negative trial.

INTRODUCTION

2a) In INTRODUCTION: Scientific background and explanation of rationale

2a-i) Problem and the type of system/solution

Describe the problem and the type of system/solution that is object of the study: intended as stand-alone intervention vs. incorporated in broader health care program? Intended for a particular patient population? Goals of the intervention, e.g., being more cost-effective to other interventions, replace or complement other solutions? (Note: Details about the intervention are provided in “Methods” under 5)

	1	2	3	4	5	
subitem not at all important	○	○	○	○	◉	essential

選択を解除

Does your paper address subitem 2a-i? *

Copy and paste relevant sections from the manuscript (include quotes in quotation marks "like this" to indicate direct quotes from your manuscript), or elaborate on this item by providing additional information not in the ms, or briefly explain why the item is not applicable/relevant for your study

”In this study, we developed a system for real-time monitoring of patient activity levels by integrating wearable devices and smartphones (Recoval © SapplyM, Inc., Tokyo, Japan). This system combines real-time data collection on physical activity and biometric metrics with online coaching to deliver personalized feedback and support to the users. The primary objectives of this pilot study were to evaluate the efficacy of this system in improving exercise capacity and adherence through continuous and interactive communication between healthcare providers and patients and to clarify the synergistic effects of online coaching on the use of wearable devices.”

2a-ii) Scientific background, rationale: What is known about the (type of) system

Scientific background, rationale: What is known about the (type of) system that is the object of the study (be sure to discuss the use of similar systems for other conditions/diagnoses, if appropiate), motivation for the study, i.e. what are the reasons for and what is the context for this specific study, from which stakeholder viewpoint is the study performed, potential impact of findings [2]. Briefly justify the choice of the comparator.

	1	2	3	4	5	
subitem not at all important	○	○	○	○	◉	essential

選択を解除

Does your paper address subitem 2a-ii? *

Copy and paste relevant sections from the manuscript (include quotes in quotation marks "like this" to indicate direct quotes from your manuscript), or elaborate on this item by providing additional information not in the ms, or briefly explain why the item is not applicable/relevant for your study

”In this study, we developed a system for real-time monitoring of patient activity levels by integrating wearable devices and smartphones (Recoval © SapplyM, Inc., Tokyo, Japan). This system combines real-time data collection on physical activity and biometric metrics with online coaching to deliver personalized feedback and support to the users. The primary objectives of this pilot study were to evaluate the efficacy of this system in improving exercise capacity and adherence through continuous and interactive communication between healthcare providers and patients and to clarify the synergistic effects of online coaching on the use of wearable devices. We hypothesized that the combination of wearable devices and online coaching would result in greater improvements in exercise capacity and adherence than those achieved through the use of wearable devices alone. ”

2b) In INTRODUCTION: Specific objectives or hypotheses

Does your paper address CONSORT subitem 2b? *

Copy and paste relevant sections from the manuscript (include quotes in quotation marks "like this" to indicate direct quotes from your manuscript), or elaborate on this item by providing additional information not in the ms, or briefly explain why the item is not applicable/relevant for your study

”We hypothesized that the combination of wearable devices and online coaching would result in greater improvements in exercise capacity and adherence than those achieved through the use of wearable devices alone. ”

METHODS

3a) Description of trial design (such as parallel, factorial) including allocation ratio

Does your paper address CONSORT subitem 3a? *

Copy and paste relevant sections from the manuscript (include quotes in quotation marks "like this" to indicate direct quotes from your manuscript), or elaborate on this item by providing additional information not in the ms, or briefly explain why the item is not applicable/relevant for your study

”This was a randomised, open-label, comparative, parallel-group, single-centre interventional study.”

3b) Important changes to methods after trial commencement (such as eligibility criteria), with reasons

Does your paper address CONSORT subitem 3b? *

Copy and paste relevant sections from the manuscript (include quotes in quotation marks "like this" to indicate direct quotes from your manuscript), or elaborate on this item by providing additional information not in the ms, or briefly explain why the item is not applicable/relevant for your study

There were no important changes in our study.

3b-i) Bug fixes, Downtimes, Content Changes

Bug fixes, Downtimes, Content Changes: ehealth systems are often dynamic systems. A description of changes to methods therefore also includes important changes made on the intervention or comparator during the trial (e.g., major bug fixes or changes in the functionality or content) (5-iii) and other “unexpected events” that may have influenced study design such as staff changes, system failures/downtimes, etc. [2].

	1	2	3	4	5	
subitem not at all important	○	○	◉	○	○	essential

選択を解除

Does your paper address subitem 3b-i?

Copy and paste relevant sections from the manuscript (include quotes in quotation marks "like this" to indicate direct quotes from your manuscript), or elaborate on this item by providing additional information not in the ms, or briefly explain why the item is not applicable/relevant for your study

There were no important changes in our study.

4a) Eligibility criteria for participants

Does your paper address CONSORT subitem 4a? *

Copy and paste relevant sections from the manuscript (include quotes in quotation marks "like this" to indicate direct quotes from your manuscript), or elaborate on this item by providing additional information not in the ms, or briefly explain why the item is not applicable/relevant for your study

"The Ethics Committee of the Juntendo Clinical Research and Trial Centre approved this study (IRB number E21-0353). Patients were enrolled by outpatient primary care physicians who were not involved in the analysis, and external clinical research coordinators allocated them into the two groups. All patients provided written informed consent to participate in the study and had the option to opt out of the study at their discretion. The collected data was de-identified and managed accordingly. Additionally, no compensation was provided to study participants."

4a-i) Computer / Internet literacy

Computer / Internet literacy is often an implicit “de facto” eligibility criterion - this should be explicitly clarified.

	1	2	3	4	5	
subitem not at all important	○	○	○	○	●	essential

選択を解除

Does your paper address subitem 4a-i?

Copy and paste relevant sections from the manuscript (include quotes in quotation marks "like this" to indicate direct quotes from your manuscript), or elaborate on this item by providing additional information not in the ms, or briefly explain why the item is not applicable/relevant for your study

”This study had some limitations. First, there was a potential selection bias in the cases enrolled in this study, as it may have included many individuals with high health consciousness and tolerance for wearable devices and desire for online coaching. These factors may limit the generalizability of our findings to population groups with lower levels of health consciousness or technological proficiency.”

4a-ii) Open vs. closed, web-based vs. face-to-face assessments:

Open vs. closed, web-based vs. face-to-face assessments: Mention how participants were recruited (online vs. offline), e.g., from an open access website or from a clinic, and clarify if this was a purely web-based trial, or there were face-to-face components (as part of the intervention or for assessment), i.e., to what degree got the study team to know the participant. In online-only trials, clarify if participants were quasi-anonymous and whether having multiple identities was possible or whether technical or logistical measures (e.g., cookies, email confirmation, phone calls) were used to detect/prevent these.

	1	2	3	4	5	
subitem not at all important	○	○	○	○	◉	essential

選択を解除

Does your paper address subitem 4a-ii? *

Copy and paste relevant sections from the manuscript (include quotes in quotation marks "like this" to indicate direct quotes from your manuscript), or elaborate on this item by providing additional information not in the ms, or briefly explain why the item is not applicable/relevant for your study

”We enrolled 50 patients with CAD who were eligible for outpatient CR (phase III) for the secondary prevention of CAD at Juntendo University Hospital between April 30, 2022 and January 21, 2023. ”

4a-iii) Information giving during recruitment

Information given during recruitment. Specify how participants were briefed for recruitment and in the informed consent procedures (e.g., publish the informed consent documentation as appendix, see also item X26), as this information may have an effect on user self-selection, user expectation and may also bias results.

	1	2	3	4	5	
subitem not at all important	○	○	○	○	◉	essential

選択を解除

Does your paper address subitem 4a-iii?

Copy and paste relevant sections from the manuscript (include quotes in quotation marks "like this" to indicate direct quotes from your manuscript), or elaborate on this item by providing additional information not in the ms, or briefly explain why the item is not applicable/relevant for your study

”The smartphones used by the patients were personal devices, and the wearable device was a Fitbit Sense (Fitbit, Inc., California, United States). Patient data were collected using a wearable device linked to a smartphone for real-time monitoring of step count, activity level, and heart rate (Recoval © SapplyM, Inc., Tokyo, Japan. For detailed information about the product, please visit the following URL: M3 Inc. "Recoval." Accessed June 25, 2024. https://m3comlp.m3.com/lp/sapplym/recoval.).”
”All patients provided written informed consent to participate in the study and had the option to opt out of the study at their discretion. ”
”Additionally, no compensation was provided to study participants.”

4b) Settings and locations where the data were collected

Does your paper address CONSORT subitem 4b? *

Copy and paste relevant sections from the manuscript (include quotes in quotation marks "like this" to indicate direct quotes from your manuscript), or elaborate on this item by providing additional information not in the ms, or briefly explain why the item is not applicable/relevant for your study

”The collected data was de-identified and managed accordingly.”

4b-i) Report if outcomes were (self-)assessed through online questionnaires

Clearly report if outcomes were (self-)assessed through online questionnaires (as common in web-based trials) or otherwise.

	1	2	3	4	5	
subitem not at all important	○	○	○	○	●	essential

選択を解除

Does your paper address subitem 4b-i? *

Copy and paste relevant sections from the manuscript (include quotes in quotation marks "like this" to indicate direct quotes from your manuscript), or elaborate on this item by providing additional information not in the ms, or briefly explain why the item is not applicable/relevant for your study

”Anxiety levels were assessed using a self-administered State Trait Anxiety Inventory (STAI) form at baseline and at the end of the study. This inventory consisted of 40 statements about the feelings of the participants and was divided into two parts. In Part I (comprising 20 statements), patients were instructed to rate the intensity of their current feelings of anxiety (indicating state anxiety) on a scale ranging from 1 (absolutely not) to 4 (very much). In Part II (the remaining 20 statements), patients reported the frequency of their general symptoms of anxiety (indicating trait anxiety) on a scale ranging from 1 (hardly ever) to 4 (often). The total score for each part ranges from 20 to 80, with higher scores indicating higher levels of anxiety. The Japanese version of the STAI was used in this study. Health-related quality of life (HR-QOL) was assessed using the Japanese version of the 36-Item short form health survey (SF-36) at baseline and at the end of the study [21, 22]. SF-36 measures eight health domains: physical function, physical role, body pain, general health, vitality, social function, emotional role, and mental health. Each domain was scored separately from 0 (indicating the lowest level of functioning) to 100 (the highest level).”

4b-ii) Report how institutional affiliations are displayed

Report how institutional affiliations are displayed to potential participants [on ehealth media], as affiliations with prestigious hospitals or universities may affect volunteer rates, use, and reactions with regards to an intervention.(Not a required item – describe only if this may bias results)

	1	2	3	4	5	
subitem not at all important	○	○	○	○	●	essential

選択を解除

Does your paper address subitem 4b-ii?

Copy and paste relevant sections from the manuscript (include quotes in quotation marks "like this" to indicate direct quotes from your manuscript), or elaborate on this item by providing additional information not in the ms, or briefly explain why the item is not applicable/relevant for your study

”Juntendo University Hospital”

5) The interventions for each group with sufficient details to allow replication, including how and when they were actually administered

5-i) Mention names, credential, affiliations of the developers, sponsors, and owners

Mention names, credential, affiliations of the developers, sponsors, and owners [6] (if authors/evaluators are owners or developer of the software, this needs to be declared in a “Conflict of interest” section or mentioned elsewhere in the manuscript).

	1	2	3	4	5	
subitem not at all important	○	○	○	○	●	essential

選択を解除

Does your paper address subitem 5-i?

Copy and paste relevant sections from the manuscript (include quotes in quotation marks "like this" to indicate direct quotes from your manuscript), or elaborate on this item by providing additional information not in the ms, or briefly explain why the item is not applicable/relevant for your study

”The intervention lasted for 12 weeks, and all patients underwent a cardiopulmonary exercise test (CPET)-based home exercise program. This program aims to engage in exercise at AT heart rate and a Borg scale of 12–13, for at least 30 min per day, five days a week. Additionally, the patients in the OLC group were provided with exercise guidance based on their activity levels recorded by their wearable device. Guidance was provided via weekly text messages and monthly video conferences. The participants in the CON group wore only the wearable device and did not receive any additional instructions (Figure 1). ”

5-ii) Describe the history/development process

Describe the history/development process of the application and previous formative evaluations (e.g., focus groups, usability testing), as these will have an impact on adoption/use rates and help with interpreting results.

	1	2	3	4	5	
subitem not at all important	○	○	○	○	●	essential

選択を解除

Does your paper address subitem 5-ii?

Copy and paste relevant sections from the manuscript (include quotes in quotation marks "like this" to indicate direct quotes from your manuscript), or elaborate on this item by providing additional information not in the ms, or briefly explain why the item is not applicable/relevant for your study

”The smartphones used by the patients were personal devices, and the wearable device was a Fitbit Sense (Fitbit, Inc., California, United States). Patient data were collected using a wearable device linked to a smartphone for real-time monitoring of step count, activity level, and heart rate (Recoval © SapplyM, Inc., Tokyo, Japan. For detailed information about the product, please visit the following URL: M3 Inc. "Recoval." Accessed June 25, 2024. https://m3comlp.m3.com/lp/sapplym/recoval.). A proprietary algorithm converted the acceleration signals from a wearable device into step counts and metabolic-equivalent tasks (METs). ”

5-iii) Revisions and updating

Revisions and updating. Clearly mention the date and/or version number of the application/intervention (and comparator, if applicable) evaluated, or describe whether the intervention underwent major changes during the evaluation process, or whether the development and/or content was “frozen” during the trial. Describe dynamic components such as news feeds or changing content which may have an impact on the replicability of the intervention (for unexpected events see item 3b).

	1	2	3	4	5	
subitem not at all important	○	○	○	○	◉	essential

選択を解除

Does your paper address subitem 5-iii?

Copy and paste relevant sections from the manuscript (include quotes in quotation marks "like this" to indicate direct quotes from your manuscript), or elaborate on this item by providing additional information not in the ms, or briefly explain why the item is not applicable/relevant for your study

”Recoval." Accessed June 25, 2024. https://m3comlp.m3.com/lp/sapplym/recoval.”
There were no major changes to the application used in our study.

5-iv) Quality assurance methods

Provide information on quality assurance methods to ensure accuracy and quality of information provided [1], if applicable.

	1	2	3	4	5	
subitem not at all important	○	○	○	○	◉	essential

選択を解除

Does your paper address subitem 5-iv?

Copy and paste relevant sections from the manuscript (include quotes in quotation marks "like this" to indicate direct quotes from your manuscript), or elaborate on this item by providing additional information not in the ms, or briefly explain why the item is not applicable/relevant for your study

”The Recoval © system provides three specific functionalities: (1) visualization of activity level and vital signs during use, (2) personalized activity goal setting, and (3) message-based intervention. The system enables medical personnel to remotely monitor patients' activity and vital data, including heart rate, steps, and calories burned, through data acquired from supported wearable devices. This data can be visualized on both the medical personnel’s and the patient’s devices, allowing for continuous tracking and analysis. Medical personnel can set individualized exercise goals for each patient, such as target exercise duration, target days per week, target heart rate during exercise, maximum allowable heart rate, and target step count per day. Patients can view these personalized goals and track their progress. Furthermore, the system provides a weekly summary that shows the number of target days versus actual days achieved, as well as comparison of actual step counts to daily targets. The system also facilitates asynchronous communication between patients and medical personnel via a dedicated message screen, where medical personnel can provide tailored feedback, guidance, and motivational messages, while patients can report their conditions and concerns in real time. This communication fosters continuous interaction, enhancing patient engagement and adherence (Supplemental material).”

5-v) Ensure replicability by publishing the source code, and/or providing screenshots/screen-capture video, and/or providing flowcharts of the algorithms used

Ensure replicability by publishing the source code, and/or providing screenshots/screen-capture video, and/or providing flowcharts of the algorithms used. Replicability (i.e., other researchers should in principle be able to replicate the study) is a hallmark of scientific reporting.

	1	2	3	4	5	
subitem not at all important	○	○	○	○	◉	essential

選択を解除

Does your paper address subitem 5-v?

Copy and paste relevant sections from the manuscript (include quotes in quotation marks "like this" to indicate direct quotes from your manuscript), or elaborate on this item by providing additional information not in the ms, or briefly explain why the item is not applicable/relevant for your study

”The Recoval © system provides three specific functionalities: (1) visualization of activity level and vital signs during use, (2) personalized activity goal setting, and (3) message-based intervention. The system enables medical personnel to remotely monitor patients' activity and vital data, including heart rate, steps, and calories burned, through data acquired from supported wearable devices. This data can be visualized on both the medical personnel’s and the patient’s devices, allowing for continuous tracking and analysis. Medical personnel can set individualized exercise goals for each patient, such as target exercise duration, target days per week, target heart rate during exercise, maximum allowable heart rate, and target step count per day. Patients can view these personalized goals and track their progress. Furthermore, the system provides a weekly summary that shows the number of target days versus actual days achieved, as well as comparison of actual step counts to daily targets. The system also facilitates asynchronous communication between patients and medical personnel via a dedicated message screen, where medical personnel can provide tailored feedback, guidance, and motivational messages, while patients can report their conditions and concerns in real time. This communication fosters continuous interaction, enhancing patient engagement and adherence (Supplemental material).”

5-vi) Digital preservation

Digital preservation: Provide the URL of the application, but as the intervention is likely to change or disappear over the course of the years; also make sure the intervention is archived (Internet Archive, webcitation.org, and/or publishing the source code or screenshots/videos alongside the article). As pages behind login screens cannot be archived, consider creating demo pages which are accessible without login.

	1	2	3	4	5	
subitem not at all important	○	○	○	○	◉	essential

選択を解除

Does your paper address subitem 5-vi?

Copy and paste relevant sections from the manuscript (include quotes in quotation marks "like this" to indicate direct quotes from your manuscript), or elaborate on this item by providing additional information not in the ms, or briefly explain why the item is not applicable/relevant for your study

”Recoval." Accessed June 25, 2024. https://m3comlp.m3.com/lp/sapplym/recoval.”
”The Recoval © system provides three specific functionalities: (1) visualization of activity level and vital signs during use, (2) personalized activity goal setting, and (3) message-based intervention. The system enables medical personnel to remotely monitor patients' activity and vital data, including heart rate, steps, and calories burned, through data acquired from supported wearable devices. This data can be visualized on both the medical personnel’s and the patient’s devices, allowing for continuous tracking and analysis. Medical personnel can set individualized exercise goals for each patient, such as target exercise duration, target days per week, target heart rate during exercise, maximum allowable heart rate, and target step count per day. Patients can view these personalized goals and track their progress. Furthermore, the system provides a weekly summary that shows the number of target days versus actual days achieved, as well as comparison of actual step counts to daily targets. The system also facilitates asynchronous communication between patients and medical personnel via a dedicated message screen, where medical personnel can provide tailored feedback, guidance, and motivational messages, while patients can report their conditions and concerns in real time. This communication fosters continuous interaction, enhancing patient engagement and adherence (Supplemental material).”

5-vii) Access

Access: Describe how participants accessed the application, in what setting/context, if they had to pay (or were paid) or not, whether they had to be a member of specific group. If known, describe how participants obtained “access to the platform and Internet” [1]. To ensure access for editors/reviewers/readers, consider to provide a “backdoor” login account or demo mode for reviewers/readers to explore the application (also important for archiving purposes, see vi).

	1	2	3	4	5	
subitem not at all important	○	○	○	○	◉	essential

選択を解除

Does your paper address subitem 5-vii? *

Copy and paste relevant sections from the manuscript (include quotes in quotation marks "like this" to indicate direct quotes from your manuscript), or elaborate on this item by providing additional information not in the ms, or briefly explain why the item is not applicable/relevant for your study

” The smartphones used by the patients were personal devices”
”Additionally, no compensation was provided to study participants.”

5-viii) Mode of delivery, features/functionalities/components of the intervention and comparator, and the theoretical framework

Describe mode of delivery, features/functionalities/components of the intervention and comparator, and the theoretical framework [6] used to design them (instructional strategy [1], behaviour change techniques, persuasive features, etc., see e.g., [7, 8] for terminology). This includes an in-depth description of the content (including where it is coming from and who developed it) [1],” whether [and how] it is tailored to individual circumstances and allows users to track their progress and receive feedback” [6]. This also includes a description of communication delivery channels and – if computer-mediated communication is a component – whether communication was synchronous or asynchronous [6]. It also includes information on presentation strategies [1], including page design principles, average amount of text on pages, presence of hyperlinks to other resources, etc. [1].

	1	2	3	4	5	
subitem not at all important	○	○	○	○	◉	essential

選択を解除

Does your paper address subitem 5-viii? *

Copy and paste relevant sections from the manuscript (include quotes in quotation marks "like this" to indicate direct quotes from your manuscript), or elaborate on this item by providing additional information not in the ms, or briefly explain why the item is not applicable/relevant for your study

” The intervention lasted for 12 weeks, and all patients underwent a cardiopulmonary exercise test (CPET)-based home exercise program. This program aims to engage in exercise at AT heart rate and a Borg scale of 12–13, for at least 30 min per day, five days a week. Additionally, the patients in the OLC group were provided with exercise guidance based on their activity levels recorded by their wearable device. Guidance was provided via weekly text messages and monthly video conferences. The participants in the CON group wore only the wearable device and did not receive any additional instructions (Figure 1). ”

5-ix) Describe use parameters

Describe use parameters (e.g., intended “doses” and optimal timing for use). Clarify what instructions or recommendations were given to the user, e.g., regarding timing, frequency, heaviness of use, if any, or was the intervention used ad libitum.

	1	2	3	4	5	
subitem not at all important	○	○	○	○	◉	essential

選択を解除

Does your paper address subitem 5-ix?

Copy and paste relevant sections from the manuscript (include quotes in quotation marks "like this" to indicate direct quotes from your manuscript), or elaborate on this item by providing additional information not in the ms, or briefly explain why the item is not applicable/relevant for your study

” The intervention lasted for 12 weeks, and all patients underwent a cardiopulmonary exercise test (CPET)-based home exercise program. This program aims to engage in exercise at AT heart rate and a Borg scale of 12–13, for at least 30 min per day, five days a week. Additionally, the patients in the OLC group were provided with exercise guidance based on their activity levels recorded by their wearable device. Guidance was provided via weekly text messages and monthly video conferences. The participants in the CON group wore only the wearable device and did not receive any additional instructions (Figure 1). ”

5-x) Clarify the level of human involvement

Clarify the level of human involvement (care providers or health professionals, also technical assistance) in the e-intervention or as co-intervention (detail number and expertise of professionals involved, if any, as well as “type of assistance offered, the timing and frequency of the support, how it is initiated, and the medium by which the assistance is delivered”. It may be necessary to distinguish between the level of human involvement required for the trial, and the level of human involvement required for a routine application outside of a RCT setting (discuss under item 21 – generalizability).

	1	2	3	4	5	
subitem not at all important	○	○	○	○	●	essential

選択を解除

Does your paper address subitem 5-x?

Copy and paste relevant sections from the manuscript (include quotes in quotation marks "like this" to indicate direct quotes from your manuscript), or elaborate on this item by providing additional information not in the ms, or briefly explain why the item is not applicable/relevant for your study

” The intervention lasted for 12 weeks, and all patients underwent a cardiopulmonary exercise test (CPET)-based home exercise program. This program aims to engage in exercise at AT heart rate and a Borg scale of 12–13, for at least 30 min per day, five days a week. Additionally, the patients in the OLC group were provided with exercise guidance based on their activity levels recorded by their wearable device. Guidance was provided via weekly text messages and monthly video conferences. The participants in the CON group wore only the wearable device and did not receive any additional instructions (Figure 1). ”

5-xi) Report any prompts/reminders used

Report any prompts/reminders used: Clarify if there were prompts (letters, emails, phone calls, SMS) to use the application, what triggered them, frequency etc. It may be necessary to distinguish between the level of prompts/reminders required for the trial, and the level of prompts/reminders for a routine application outside of a RCT setting (discuss under item 21 – generalizability).

	1	2	3	4	5	
subitem not at all important	○	○	○	○	◉	essential

選択を解除

Does your paper address subitem 5-xi? *

Copy and paste relevant sections from the manuscript (include quotes in quotation marks "like this" to indicate direct quotes from your manuscript), or elaborate on this item by providing additional information not in the ms, or briefly explain why the item is not applicable/relevant for your study

”Additionally, the patients in the OLC group were provided with exercise guidance based on their activity levels recorded by their wearable device. Guidance was provided via weekly text messages and monthly video conferences. The participants in the CON group wore only the wearable device and did not receive any additional instructions (Figure 1). ”

5-xii) Describe any co-interventions (incl. training/support)

Describe any co-interventions (incl. training/support): Clearly state any interventions that are provided in addition to the targeted eHealth intervention, as ehealth intervention may not be designed as stand-alone intervention. This includes training sessions and support [1]. It may be necessary to distinguish between the level of training required for the trial, and the level of training for a routine application outside of a RCT setting (discuss under item 21 – generalizability.

	1	2	3	4	5	
subitem not at all important	○	○	○	○	◉	essential

選択を解除

Does your paper address subitem 5-xii? *

Copy and paste relevant sections from the manuscript (include quotes in quotation marks "like this" to indicate direct quotes from your manuscript), or elaborate on this item by providing additional information not in the ms, or briefly explain why the item is not applicable/relevant for your study

”Additionally, the patients in the OLC group were provided with exercise guidance based on their activity levels recorded by their wearable device. Guidance was provided via weekly text messages and monthly video conferences. The participants in the CON group wore only the wearable device and did not receive any additional instructions (Figure 1). ”

6a) Completely defined pre-specified primary and secondary outcome measures, including how and when they were assessed

Does your paper address CONSORT subitem 6a? *

Copy and paste relevant sections from the manuscript (include quotes in quotation marks "like this" to indicate direct quotes from your manuscript), or elaborate on this item by providing additional information not in the ms, or briefly explain why the item is not applicable/relevant for your study

”We assessed the anthropometric parameters of patients and exercise tolerance at the beginning and end of the study. Briefly, anthropometric parameters, including the percentage of body fat, lean body weight, and muscle mass, were measured using bioelectrical impedance analysis (MC-780A; TANITA, Tokyo, Japan). The bioelectrical impedance analysis measurements were conducted 2–3 hours after a meal and prior to CPET. Exercise capacity was assessed using CPET on a cycle ergometer (Strength Ergo 8, MITSUBISHI ELECTRIC, Tokyo, Japan) with an expiratory gas analysis machine (AE-310S, MINATO MEDICAL SCIENCE Co., Ltd., Osaka, Japan). After 4 min of rest in the sitting position, the patient warmed up for several minutes at 20 W, followed by ramp loading (10 W/min) until they felt exhausted or experienced progressive angina, ST-segment depression (≥2 mm), or sustained tachyarrhythmia [18]. A standard 12-lead electrocardiogram was continuously recorded, and heart rate and BP were recorded every minute during the exercise test. A satisfactory endpoint of CPET was a respiratory exchange ratio of greater than 1.10 [19]. Peak oxygen consumption (peak VO2) was defined as the highest VO2 value recorded during CPET, and the anaerobic threshold (AT) point was determined by the “V-slope” method [20].
Anxiety levels were assessed using a self-administered State Trait Anxiety Inventory (STAI) form at baseline and at the end of the study. This inventory consisted of 40 statements about the feelings of the participants and was divided into two parts. In Part I (comprising 20 statements), patients were instructed to rate the intensity of their current feelings of anxiety (indicating state anxiety) on a scale ranging from 1 (absolutely not) to 4 (very much). In Part II (the remaining 20 statements), patients reported the frequency of their general symptoms of anxiety (indicating trait anxiety) on a scale ranging from 1 (hardly ever) to 4 (often). The total score for each part ranges from 20 to 80, with higher scores indicating higher levels of anxiety. The Japanese version of the STAI was used in this study. Health-related quality of life (HR-QOL) was assessed using the Japanese version of the 36-Item short form health survey (SF-36) at baseline and at the end of the study [21, 22]. SF-36 measures eight health domains: physical function, physical role, body pain, general health, vitality, social function, emotional role, and mental health. Each domain was scored separately from 0 (indicating the lowest level of functioning) to 100 (the highest level).
Activity level was categorised as sedentary (<1.5 METs), lightly active (1.5–3 METs), moderately active (3–6 METs), or highly active (>6 METs or ≥145 steps/min sustained for at least 10 min).
”
”The primary outcomes for this study included changes in peak VO2 and AT VO2 at 12 weeks. The secondary outcomes for this study were changes in CPET parameters, daily activity, anxiety level, and HR-QOL.”

6a-i) Online questionnaires: describe if they were validated for online use and apply CHERRIES items to describe how the questionnaires were designed/deployed

If outcomes were obtained through online questionnaires, describe if they were validated for online use and apply CHERRIES items to describe how the questionnaires were designed/deployed [9].

	1	2	3	4	5	
subitem not at all important	○	○	◉	○	○	essential

選択を解除

Does your paper address subitem 6a-i?

Copy and paste relevant sections from manuscript text

We did not use online questionnaires in our study.

6a-ii) Describe whether and how “use” (including intensity of use/dosage) was defined/measured/monitored

Describe whether and how “use” (including intensity of use/dosage) was defined/measured/monitored (logins, logfile analysis, etc.). Use/adoption metrics are important process outcomes that should be reported in any ehealth trial.

	1	2	3	4	5	
subitem not at all important	○	○	○	○	◉	essential

選択を解除

Does your paper address subitem 6a-ii?

Copy and paste relevant sections from manuscript text

”the wearable device was a Fitbit Sense (Fitbit, Inc., California, United States). Patient data were collected using a wearable device linked to a smartphone for real-time monitoring of step count, activity level, and heart rate (Recoval © SapplyM, Inc., Tokyo, Japan. For detailed information about the product, please visit the following URL: M3 Inc. "Recoval." ”

6a-iii) Describe whether, how, and when qualitative feedback from participants was obtained

Describe whether, how, and when qualitative feedback from participants was obtained (e.g., through emails, feedback forms, interviews, focus groups).

	1	2	3	4	5	
subitem not at all important	○	○	○	●	○	essential

選択を解除

Does your paper address subitem 6a-iii?

Copy and paste relevant sections from manuscript text

”Anxiety levels were assessed using a self-administered State Trait Anxiety Inventory (STAI) form at baseline and at the end of the study. This inventory consisted of 40 statements about the feelings of the participants and was divided into two parts. In Part I (comprising 20 statements), patients were instructed to rate the intensity of their current feelings of anxiety (indicating state anxiety) on a scale ranging from 1 (absolutely not) to 4 (very much). In Part II (the remaining 20 statements), patients reported the frequency of their general symptoms of anxiety (indicating trait anxiety) on a scale ranging from 1 (hardly ever) to 4 (often). The total score for each part ranges from 20 to 80, with higher scores indicating higher levels of anxiety. The Japanese version of the STAI was used in this study. Health-related quality of life (HR-QOL) was assessed using the Japanese version of the 36-Item short form health survey (SF-36) at baseline and at the end of the study [21, 22]. SF-36 measures eight health domains: physical function, physical role, body pain, general health, vitality, social function, emotional role, and mental health. Each domain was scored separately from 0 (indicating the lowest level of functioning) to 100 (the highest level).”

6b) Any changes to trial outcomes after the trial commenced, with reasons

Does your paper address CONSORT subitem 6b? *

Copy and paste relevant sections from the manuscript (include quotes in quotation marks "like this" to indicate direct quotes from your manuscript), or elaborate on this item by providing additional information not in the ms, or briefly explain why the item is not applicable/relevant for your study

There were no changes to the trial outcomes after commencement.

7a) How sample size was determined

NPT: When applicable, details of whether and how the clustering by care provides or centers was addressed

7a-i) Describe whether and how expected attrition was taken into account when calculating the sample size

Describe whether and how expected attrition was taken into account when calculating the sample size.

	1	2	3	4	5	
subitem not at all important	○	○	○	○	◉	essential

選択を解除

Does your paper address subitem 7a-i?

Copy and paste relevant sections from manuscript title (include quotes in quotation marks "like this" to indicate direct quotes from your manuscript), or elaborate on this item by providing additional information not in the ms, or briefly explain why the item is not applicable/relevant for your study

"The sample size calculation was based on anticipated changes in peak VO2 between the OLC and CON groups, as reportedly previously by comparable studies. We assumed a mean improvement in peak VO2 of 2.0 mL/kg/min in the OLC group and 1.0 mL/kg/min in the CON group, estimating this as approximately 80% of the peak VO2 increase observed in comparable studies [23]. To determine the sample size, we used a pooled standard deviation of 1.1 mL/kg/min, a two-sided significance level of 5%, and a power of 80%, which led to an initial requirement of 22 participants per group. To account for an anticipated 10% dropout rate, we adjusted the final sample size to 25 participants per group."

7b) When applicable, explanation of any interim analyses and stopping guidelines

Does your paper address CONSORT subitem 7b? *

Copy and paste relevant sections from the manuscript (include quotes in quotation marks "like this" to indicate direct quotes from your manuscript), or elaborate on this item by providing additional information not in the ms, or briefly explain why the item is not applicable/relevant for your study

No interim analyses or stopping guidelines were planned.

8a) Method used to generate the random allocation sequence
NPT: When applicable, how care providers were allocated to each trial group

Does your paper address CONSORT subitem 8a? *

Copy and paste relevant sections from the manuscript (include quotes in quotation marks "like this" to indicate direct quotes from your manuscript), or elaborate on this item by providing additional information not in the ms, or briefly explain why the item is not applicable/relevant for your study

”In this study, patients were randomly divided into a wearable device plus online coaching group (OLC) and a control wearable device group (CON), stratified by age (<60 years and ≥60 years) and sex (male and female), using a stratified permuted block design as the allocation method. ”

8b) Type of randomisation; details of any restriction (such as blocking and block size)

Does your paper address CONSORT subitem 8b? *

Copy and paste relevant sections from the manuscript (include quotes in quotation marks "like this" to indicate direct quotes from your manuscript), or elaborate on this item by providing additional information not in the ms, or briefly explain why the item is not applicable/relevant for your study

”In this study, patients were randomly divided into a wearable device plus online coaching group (OLC) and a control wearable device group (CON), stratified by age (<60 years and ≥60 years) and sex (male and female), using a stratified permuted block design as the allocation method. ”

9) Mechanism used to implement the random allocation sequence (such as sequentially numbered containers), describing any steps taken to conceal the sequence until interventions were assigned

Does your paper address CONSORT subitem 9? *

Copy and paste relevant sections from the manuscript (include quotes in quotation marks "like this" to indicate direct quotes from your manuscript), or elaborate on this item by providing additional information not in the ms, or briefly explain why the item is not applicable/relevant for your study

”Patients were enrolled by outpatient primary care physicians who were not involved in the analysis, and external clinical research coordinators allocated them into the two groups. ”
”Mechanism used to implement the random allocation sequence (such as sequentially numbered containers), describing any steps taken to conceal the sequence until interventions were assigned”
”The collected data was de-identified and managed accordingly. ”

10) Who generated the random allocation sequence, who enrolled participants, and who assigned participants to interventions

Does your paper address CONSORT subitem 10? *

Copy and paste relevant sections from the manuscript (include quotes in quotation marks "like this" to indicate direct quotes from your manuscript), or elaborate on this item by providing additional information not in the ms, or briefly explain why the item is not applicable/relevant for your study

”Patients were enrolled by outpatient primary care physicians who were not involved in the analysis, and external clinical research coordinators allocated them into the two groups. ”

11a) If done, who was blinded after assignment to interventions (for example, participants, care providers, those assessing outcomes) and how

NPT: Whether or not administering co-interventions were blinded to group assignment

11a-i) Specify who was blinded, and who wasn’t

Specify who was blinded, and who wasn’t. Usually, in web-based trials it is not possible to blind the participants [1, 3] (this should be clearly acknowledged), but it may be possible to blind outcome assessors, those doing data analysis or those administering co-interventions (if any).

	1	2	3	4	5	
subitem not at all important	○	○	○	○	◉	essential

選択を解除

Does your paper address subitem 11a-i? *

Copy and paste relevant sections from the manuscript (include quotes in quotation marks "like this" to indicate direct quotes from your manuscript), or elaborate on this item by providing additional information not in the ms, or briefly explain why the item is not applicable/relevant for your study

”This was a randomised, open-label, comparative, parallel-group, single-centre interventional study. ”
”Patients were enrolled by outpatient primary care physicians who were not involved in the analysis, and external clinical research coordinators allocated them into the two groups. ”
”The collected data was de-identified and managed accordingly. ”

11a-ii) Discuss e.g., whether participants knew which intervention was the "intervention of interest" and which one was the "comparator"

Informed consent procedures (4a-ii) can create biases and certain expectations - discuss e.g., whether participants knew which intervention was the "intervention of interest" and which one was the "comparator".

	1	2	3	4	5	
subitem not at all important	○	○	○	○	◉	essential

選択を解除

Does your paper address subitem 11a-ii?

Copy and paste relevant sections from the manuscript (include quotes in quotation marks "like this" to indicate direct quotes from your manuscript), or elaborate on this item by providing additional information not in the ms, or briefly explain why the item is not applicable/relevant for your study

"The intervention lasted for 12 weeks, and all patients underwent a cardiopulmonary exercise test (CPET)-based home exercise program. This program aims to engage in exercise at AT heart rate and a Borg scale of 12–13, for at least 30 min per day, five days a week. Additionally, the patients in the OLC group were provided with exercise guidance based on their activity levels recorded by their wearable device. Guidance was provided via weekly text messages and monthly video conferences. The participants in the CON group wore only the wearable device and did not receive any additional instructions"

11b) If relevant, description of the similarity of interventions

(this item is usually not relevant for ehealth trials as it refers to similarity of a placebo or sham intervention to a active medication/intervention)

Does your paper address CONSORT subitem 11b? *

Copy and paste relevant sections from the manuscript (include quotes in quotation marks "like this" to indicate direct quotes from your manuscript), or elaborate on this item by providing additional information not in the ms, or briefly explain why the item is not applicable/relevant for your study

This item is not applicable, as the interventions in this eHealth trial were clearly distinguishable and no placebo or sham intervention was used.

12a) Statistical methods used to compare groups for primary and secondary outcomes

NPT: When applicable, details of whether and how the clustering by care providers or centers was addressed

Does your paper address CONSORT subitem 12a? *

Copy and paste relevant sections from the manuscript (include quotes in quotation marks "like this" to indicate direct quotes from your manuscript), or elaborate on this item by providing additional information not in the ms, or briefly explain why the item is not applicable/relevant for your study

”Categorical data are presented as numbers and percentages and were compared using the chi-squared test. Continuous variables are expressed as mean ± standard deviation or median and interquartile range. Between-group comparisons were performed using Student's t-test, while within-group comparisons were performed using a paired t-test. A p-value <0.05 was considered statistically significant. Effect sizes for the group × time interaction were reported using Cohen's f, interpreted as small (f≥0.10), medium (f≥0.25), and large (f≥0.40). Missing values were not imputed, and multiplicity was not considered. In this pilot trial, the outcome assessors were blinded to group allocations. All statistical analyses were performed using R version 4.4.2 (R Foundation for Statistical Computing, Vienna, Austria).”

12a-i) Imputation techniques to deal with attrition / missing values

Imputation techniques to deal with attrition / missing values: Not all participants will use the intervention/comparator as intended and attrition is typically high in ehealth trials. Specify how participants who did not use the application or dropped out from the trial were treated in the statistical analysis (a complete case analysis is strongly discouraged, and simple imputation techniques such as LOCF may also be problematic [4]).

	1	2	3	4	5	
subitem not at all important	○	○	○	○	◉	essential

選択を解除

Does your paper address subitem 12a-i? *

Copy and paste relevant sections from the manuscript (include quotes in quotation marks "like this" to indicate direct quotes from your manuscript), or elaborate on this item by providing additional information not in the ms, or briefly explain why the item is not applicable/relevant for your study

”Missing values were not imputed, and multiplicity was not considered. ”

12b) Methods for additional analyses, such as subgroup analyses and adjusted analyses

Does your paper address CONSORT subitem 12b? *

Copy and paste relevant sections from the manuscript (include quotes in quotation marks "like this" to indicate direct quotes from your manuscript), or elaborate on this item by providing additional information not in the ms, or briefly explain why the item is not applicable/relevant for your study

No subgroup or adjusted analyses were conducted in this study.

X26) REB/IRB Approval and Ethical Considerations [recommended as subheading under "Methods"] (not a CONSORT item)

X26-i) Comment on ethics committee approval

	1	2	3	4	5	
subitem not at all important	○	○	○	○	◉	essential

選択を解除

Does your paper address subitem X26-i?

Copy and paste relevant sections from the manuscript (include quotes in quotation marks "like this" to indicate direct quotes from your manuscript), or elaborate on this item by providing additional information not in the ms, or briefly explain why the item is not applicable/relevant for your study

”The Ethics Committee of the Juntendo Clinical Research and Trial Centre approved this study (IRB number E21-0353).”

x26-ii) Outline informed consent procedures

Outline informed consent procedures e.g., if consent was obtained offline or online (how? Checkbox, etc.?), and what information was provided (see 4a-ii). See [6] for some items to be included in informed consent documents.

	1	2	3	4	5	
subitem not at all important	○	○	○	○	◉	essential

選択を解除

Does your paper address subitem X26-ii?

Copy and paste relevant sections from the manuscript (include quotes in quotation marks "like this" to indicate direct quotes from your manuscript), or elaborate on this item by providing additional information not in the ms, or briefly explain why the item is not applicable/relevant for your study

”All patients provided written informed consent to participate in the study and had the option to opt out of the study at their discretion. ”

X26-iii) Safety and security procedures

Safety and security procedures, incl. privacy considerations, and any steps taken to reduce the likelihood or detection of harm (e.g., education and training, availability of a hotline)

	1	2	3	4	5	
subitem not at all important	○	○	○	○	◉	essential

選択を解除

Does your paper address subitem X26-iii?

Copy and paste relevant sections from the manuscript (include quotes in quotation marks "like this" to indicate direct quotes from your manuscript), or elaborate on this item by providing additional information not in the ms, or briefly explain why the item is not applicable/relevant for your study

”The collected data was de-identified and managed accordingly. ”

RESULTS

13a) For each group, the numbers of participants who were randomly assigned, received intended treatment, and were analysed for the primary outcome

NPT: The number of care providers or centers performing the intervention in each group and the number of patients treated by each care provider in each center

Does your paper address CONSORT subitem 13a? *

Copy and paste relevant sections from the manuscript (include quotes in quotation marks "like this" to indicate direct quotes from your manuscript), or elaborate on this item by providing additional information not in the ms, or briefly explain why the item is not applicable/relevant for your study

”A total of 83 patients with CAD were enrolled in the study. Of these, six patients were excluded because they did not have compatible smartphones, 23 were excluded due to lack of consent, one was excluded due to uncontrolled angina, and three patients were excluded due to uncontrolled arrhythmias. As a result, 50 patients with CAD were finally analysed and classified into two groups, OLC and CON, with randomisation yielding 25 participants per group.”

13b) For each group, losses and exclusions after randomisation, together with reasons

Does your paper address CONSORT subitem 13b? (NOTE: Preferably, this is shown in a CONSORT flow diagram) *

Copy and paste relevant sections from the manuscript (include quotes in quotation marks "like this" to indicate direct quotes from your manuscript), or elaborate on this item by providing additional information not in the ms, or briefly explain why the item is not applicable/relevant for your study

”A total of 83 patients with CAD were enrolled in the study. Of these, six patients were excluded because they did not have compatible smartphones, 23 were excluded due to lack of consent, one was excluded due to uncontrolled angina, and three patients were excluded due to uncontrolled arrhythmias. As a result, 50 patients with CAD were finally analysed and classified into two groups, OLC and CON, with randomisation yielding 25 participants per group.”

13b-i) Attrition diagram

Strongly recommended: An attrition diagram (e.g., proportion of participants still logging in or using the intervention/comparator in each group plotted over time, similar to a survival curve) or other figures or tables demonstrating usage/dose/engagement.

	1	2	3	4	5	
subitem not at all important	○	○	○	○	◉	essential

選択を解除

Does your paper address subitem 13b-i?

Copy and paste relevant sections from the manuscript or cite the figure number if applicable (include quotes in quotation marks "like this" to indicate direct quotes from your manuscript), or elaborate on this item by providing additional information not in the ms, or briefly explain why the item is not applicable/relevant for your study

”One patient in the OLC group dropped out during the follow-up period because of lower limb muscle strain. ”
”Finally, baseline data and data at 12 weeks following intervention from a total of 49 patients were included in the analysis. (Supplemental Figure 1). ”

14a) Dates defining the periods of recruitment and follow-up

Does your paper address CONSORT subitem 14a? *

Copy and paste relevant sections from the manuscript (include quotes in quotation marks "like this" to indicate direct quotes from your manuscript), or elaborate on this item by providing additional information not in the ms, or briefly explain why the item is not applicable/relevant for your study

”We enrolled 50 patients with CAD who were eligible for outpatient CR (phase III) for the secondary prevention of CAD at Juntendo University Hospital between April 30, 2022 and January 21, 2023. ”

14a-i) Indicate if critical “secular events” fell into the study period

Indicate if critical “secular events” fell into the study period, e.g., significant changes in Internet resources available or “changes in computer hardware or Internet delivery resources”

	1	2	3	4	5	
subitem not at all important	○	○	◉	○	○	essential

選択を解除

Does your paper address subitem 14a-i?

Copy and paste relevant sections from the manuscript (include quotes in quotation marks "like this" to indicate direct quotes from your manuscript), or elaborate on this item by providing additional information not in the ms, or briefly explain why the item is not applicable/relevant for your study

No major secular events affecting Internet access, hardware, or health-related online resources were identified during the study period.

14b) Why the trial ended or was stopped (early)

Does your paper address CONSORT subitem 14b? *

Copy and paste relevant sections from the manuscript (include quotes in quotation marks "like this" to indicate direct quotes from your manuscript), or elaborate on this item by providing additional information not in the ms, or briefly explain why the item is not applicable/relevant for your study

The trial was completed as planned, with no early termination.

15) A table showing baseline demographic and clinical characteristics for each group

NPT: When applicable, a description of care providers (case volume, qualification, expertise, etc.) and centers (volume) in each group

Does your paper address CONSORT subitem 15? *

Copy and paste relevant sections from the manuscript (include quotes in quotation marks "like this" to indicate direct quotes from your manuscript), or elaborate on this item by providing additional information not in the ms, or briefly explain why the item is not applicable/relevant for your study

"Table 1 presents the patient backgrounds. Mean age (63.8±6.4 vs 62.6±7.9 years) and sex distribution (95.8% vs 92.0% male) between the OLC and CON groups were similar. No significant differences were observed between the two groups regarding baseline characteristics and coronary risk factors (all $p > 0.05$). "

15-i) Report demographics associated with digital divide issues

In ehealth trials it is particularly important to report demographics associated with digital divide issues, such as age, education, gender, social-economic status, computer/Internet/ehealth literacy of the participants, if known.

	1	2	3	4	5	
subitem not at all important	○	○	○	○	◉	essential

選択を解除

Does your paper address subitem 15-i? *

Copy and paste relevant sections from the manuscript (include quotes in quotation marks "like this" to indicate direct quotes from your manuscript), or elaborate on this item by providing additional information not in the ms, or briefly explain why the item is not applicable/relevant for your study

"Table 1 presents the patient backgrounds. Mean age (63.8±6.4 vs 62.6±7.9 years) and sex distribution (95.8% vs 92.0% male) between the OLC and CON groups were similar. No significant differences were observed between the two groups regarding baseline characteristics and coronary risk factors (all $p > 0.05$). "

16) For each group, number of participants (denominator) included in each analysis and whether the analysis was by original assigned groups

16-i) Report multiple “denominators” and provide definitions

Report multiple “denominators” and provide definitions: Report N’s (and effect sizes) “across a range of study participation [and use] thresholds” [1], e.g., N exposed, N consented, N used more than x times, N used more than y weeks, N participants “used” the intervention/comparator at specific pre-defined time points of interest (in absolute and relative numbers per group). Always clearly define “use” of the intervention.

	1	2	3	4	5	
subitem not at all important	○	○	○	○	◉	essential

選択を解除

Does your paper address subitem 16-i? *

Copy and paste relevant sections from the manuscript (include quotes in quotation marks "like this" to indicate direct quotes from your manuscript), or elaborate on this item by providing additional information not in the ms, or briefly explain why the item is not applicable/relevant for your study

”Finally, baseline data and data at 12 weeks following intervention from a total of 49 patients were included in the analysis. (Supplemental Figure 1). ”

16-ii) Primary analysis should be intent-to-treat

Primary analysis should be intent-to-treat, secondary analyses could include comparing only “users”, with the appropriate caveats that this is no longer a randomized sample (see 18-i).

	1	2	3	4	5	
subitem not at all important	○	○	○	○	◉	essential

選択を解除

Does your paper address subitem 16-ii?

Copy and paste relevant sections from the manuscript (include quotes in quotation marks "like this" to indicate direct quotes from your manuscript), or elaborate on this item by providing additional information not in the ms, or briefly explain why the item is not applicable/relevant for your study

” As a result, 50 patients with CAD were finally analysed and classified into two groups, OLC and CON, with randomisation yielding 25 participants per group. One patient in the OLC group dropped out during the follow-up period because of lower limb muscle strain. No major adverse cardiac events occurred during the study period; these were defined as a composite of cardiovascular death, nonfatal myocardial infarction, nonfatal stroke, and admission for heart failure. Finally, baseline data and data at 12 weeks following intervention from a total of 49 patients were included in the analysis. (Supplemental Figure 1).”

17a) For each primary and secondary outcome, results for each group, and the estimated effect size and its precision (such as 95% confidence interval)

Does your paper address CONSORT subitem 17a? *

Copy and paste relevant sections from the manuscript (include quotes in quotation marks "like this" to indicate direct quotes from your manuscript), or elaborate on this item by providing additional information not in the ms, or briefly explain why the item is not applicable/relevant for your study

”Over a period of 12 weeks, changes in CPET parameters and mental status outcomes were observed in both groups (Table 3). ”
Table3 shows the effect size.

17a-i) Presentation of process outcomes such as metrics of use and intensity of use

In addition to primary/secondary (clinical) outcomes, the presentation of process outcomes such as metrics of use and intensity of use (dose, exposure) and their operational definitions is critical. This does not only refer to metrics of attrition (13-b) (often a binary variable), but also to more continuous exposure metrics such as “average session length”. These must be accompanied by a technical description how a metric like a “session” is defined (e.g., timeout after idle time) [1] (report under item 6a).

	1	2	3	4	5	
subitem not at all important	○	○	○	○	●	essential

選択を解除

Does your paper address subitem 17a-i?

Copy and paste relevant sections from the manuscript (include quotes in quotation marks "like this" to indicate direct quotes from your manuscript), or elaborate on this item by providing additional information not in the ms, or briefly explain why the item is not applicable/relevant for your study

”The parameters measured by the wearable device are shown in Figure 2-5.”

17b) For binary outcomes, presentation of both absolute and relative effect sizes is recommended

Does your paper address CONSORT subitem 17b? *

Copy and paste relevant sections from the manuscript (include quotes in quotation marks "like this" to indicate direct quotes from your manuscript), or elaborate on this item by providing additional information not in the ms, or briefly explain why the item is not applicable/relevant for your study

Binary outcomes were not used in this study; therefore, absolute and relative effect sizes are not applicable

18) Results of any other analyses performed, including subgroup analyses and adjusted analyses, distinguishing pre-specified from exploratory

Does your paper address CONSORT subitem 18? *

Copy and paste relevant sections from the manuscript (include quotes in quotation marks "like this" to indicate direct quotes from your manuscript), or elaborate on this item by providing additional information not in the ms, or briefly explain why the item is not applicable/relevant for your study

No additional analyses, including subgroup or adjusted analyses, were performed in this study.

18-i) Subgroup analysis of comparing only users

A subgroup analysis of comparing only users is not uncommon in ehealth trials, but if done, it must be stressed that this is a self-selected sample and no longer an unbiased sample from a randomized trial (see 16-iii).

	1	2	3	4	5	
subitem not at all important	○	○	●	○	○	essential

選択を解除

Does your paper address subitem 18-i?

Copy and paste relevant sections from the manuscript (include quotes in quotation marks "like this" to indicate direct quotes from your manuscript), or elaborate on this item by providing additional information not in the ms, or briefly explain why the item is not applicable/relevant for your study

No additional analyses, including subgroup or adjusted analyses, were performed in this study.

19) All important harms or unintended effects in each group

(for specific guidance see CONSORT for harms)

Does your paper address CONSORT subitem 19? *

Copy and paste relevant sections from the manuscript (include quotes in quotation marks "like this" to indicate direct quotes from your manuscript), or elaborate on this item by providing additional information not in the ms, or briefly explain why the item is not applicable/relevant for your study

”One patient in the OLC group dropped out during the follow-up period because of lower limb muscle strain.”

19-i) Include privacy breaches, technical problems

Include privacy breaches, technical problems. This does not only include physical “harm” to participants, but also incidents such as perceived or real privacy breaches [1], technical problems, and other unexpected/unintended incidents. “Unintended effects” also includes unintended positive effects [2].

	1	2	3	4	5	
subitem not at all important	○	○	◉	○	○	essential

選択を解除

Does your paper address subitem 19-i?

Copy and paste relevant sections from the manuscript (include quotes in quotation marks "like this" to indicate direct quotes from your manuscript), or elaborate on this item by providing additional information not in the ms, or briefly explain why the item is not applicable/relevant for your study

No privacy breaches, technical issues, or other unintended incidents were reported during the course of the study.

19-ii) Include qualitative feedback from participants or observations from staff/researchers

Include qualitative feedback from participants or observations from staff/researchers, if available, on strengths and shortcomings of the application, especially if they point to unintended/unexpected effects or uses. This includes (if available) reasons for why people did or did not use the application as intended by the developers.

	1	2	3	4	5	
subitem not at all important	○	○	○	◉	○	essential

選択を解除

Does your paper address subitem 19-ii?

Copy and paste relevant sections from the manuscript (include quotes in quotation marks "like this" to indicate direct quotes from your manuscript), or elaborate on this item by providing additional information not in the ms, or briefly explain why the item is not applicable/relevant for your study

""One patient in the OLC group dropped out during the follow-up period because of lower limb muscle strain.""

DISCUSSION

22) Interpretation consistent with results, balancing benefits and harms, and considering other relevant evidence

NPT: In addition, take into account the choice of the comparator, lack of or partial blinding, and unequal expertise of care providers or centers in each group

22-i) Restate study questions and summarize the answers suggested by the data, starting with primary outcomes and process outcomes (use)

Restate study questions and summarize the answers suggested by the data, starting with primary outcomes and process outcomes (use).

	1	2	3	4	5	
subitem not at all important	○	○	○	○	◉	essential

選択を解除

Does your paper address subitem 22-i? *

Copy and paste relevant sections from the manuscript (include quotes in quotation marks "like this" to indicate direct quotes from your manuscript), or elaborate on this item by providing additional information not in the ms, or briefly explain why the item is not applicable/relevant for your study

”We investigated the effects of remote CR using wearable devices with and without online coaching in patients with CAD. The major findings of the present study were as follows: (1) using wearable devices and providing a home exercise program based on CPET for 3 months significantly improved Peak VO2 and AT VO2; (2) the OLC group demonstrated significantly higher physical activity (daily distance and daily duration of high activity) in the latter half of the intervention than did the CON group; and (3) during the research period, there were no dropouts owing to the use of wearable device or online coaching.”

22-ii) Highlight unanswered new questions, suggest future research

Highlight unanswered new questions, suggest future research.

	1	2	3	4	5	
subitem not at all important	○	○	○	○	◉	essential

選択を解除

Does your paper address subitem 22-ii?

Copy and paste relevant sections from the manuscript (include quotes in quotation marks "like this" to indicate direct quotes from your manuscript), or elaborate on this item by providing additional information not in the ms, or briefly explain why the item is not applicable/relevant for your study

” Future interventional studies with larger sample sizes, longer follow-up durations, and more diverse populations are needed to confirm the findings and evaluate long-term adherence and sustained benefits of such programs.”

20) Trial limitations, addressing sources of potential bias, imprecision, and, if relevant, multiplicity of analyses

20-i) Typical limitations in ehealth trials

Typical limitations in ehealth trials: Participants in ehealth trials are rarely blinded. Ehealth trials often look at a multiplicity of outcomes, increasing risk for a Type I error. Discuss biases due to non-use of the intervention/usability issues, biases through informed consent procedures, unexpected events.

	1	2	3	4	5	
subitem not at all important	○	○	○	○	●	essential

選択を解除

Does your paper address subitem 20-i? *

Copy and paste relevant sections from the manuscript (include quotes in quotation marks "like this" to indicate direct quotes from your manuscript), or elaborate on this item by providing additional information not in the ms, or briefly explain why the item is not applicable/relevant for your study

”This study had some limitations. First, there was a potential selection bias in the cases enrolled in this study, as it may have included many individuals with high health consciousness and tolerance for wearable devices and desire for online coaching. These factors may limit the generalizability of our findings to population groups with lower levels of health consciousness or technological proficiency. Second, this was a single-centre study conducted primarily among Japanese patients, with a small sample size and a short follow-up period of 12 weeks. Regional differences and cultural factors may have influenced the results. Furthermore, the predominance of male sex in the study cohort may also limit the applicability of the findings to females, older adults, or different ethnic groups. Additionally, varying levels of digital literacy and access to technology could impact the effectiveness of remote CR programs, both within Japan and internationally. Third, the power analysis indicates that the study is sufficiently powered to detect large effect size differences between the OLC group and CON group, specifically a Cohen's d of 0.8 with a sample size of 50. These effect sizes are rather large and may be unrealistic for the intervention being studied, suggesting that this study is underpowered for detecting more moderate or smaller effect sizes. This study serves primarily as a proof-of-concept. Future interventional studies with larger sample sizes, longer follow-up durations, and more diverse populations are needed to confirm the findings and evaluate long-term adherence and sustained benefits of such programs.”

21) Generalisability (external validity, applicability) of the trial findings

NPT: External validity of the trial findings according to the intervention, comparators, patients, and care providers or centers involved in the trial

21-i) Generalizability to other populations

Generalizability to other populations: In particular, discuss generalizability to a general Internet population, outside of a RCT setting, and general patient population, including applicability of the study results for other organizations

	1	2	3	4	5	
subitem not at all important	○	○	○	○	◉	essential

選択を解除

Does your paper address subitem 21-i?

Copy and paste relevant sections from the manuscript (include quotes in quotation marks "like this" to indicate direct quotes from your manuscript), or elaborate on this item by providing additional information not in the ms, or briefly explain why the item is not applicable/relevant for your study

” First, there was a potential selection bias in the cases enrolled in this study, as it may have included many individuals with high health consciousness and tolerance for wearable devices and desire for online coaching. These factors may limit the generalizability of our findings to population groups with lower levels of health consciousness or technological proficiency. Second, this was a single-centre study conducted primarily among Japanese patients, with a small sample size and a short follow-up period of 12 weeks. Regional differences and cultural factors may have influenced the results. Furthermore, the predominance of male sex in the study cohort may also limit the applicability of the findings to females, older adults, or different ethnic groups. Additionally, varying levels of digital literacy and access to technology could impact the effectiveness of remote CR programs, both within Japan and internationally. ”

21-ii) Discuss if there were elements in the RCT that would be different in a routine application setting

Discuss if there were elements in the RCT that would be different in a routine application setting (e.g., prompts/reminders, more human involvement, training sessions or other co-interventions) and what impact the omission of these elements could have on use, adoption, or outcomes if the intervention is applied outside of a RCT setting.

	1	2	3	4	5	
subitem not at all important	○	○	○	○	●	essential

選択を解除

Does your paper address subitem 21-ii?

Copy and paste relevant sections from the manuscript (include quotes in quotation marks "like this" to indicate direct quotes from your manuscript), or elaborate on this item by providing additional information not in the ms, or briefly explain why the item is not applicable/relevant for your study

”Discuss if there were elements in the RCT that would be different in a routine application setting
Discuss if there were elements in the RCT that would be different in a routine application setting (e.g., prompts/reminders, more human involvement, training sessions or other co-interventions) and what impact the omission of these elements could have on use, adoption, or outcomes if the intervention is applied outside of a RCT setting.”
”Future interventional studies with larger sample sizes, longer follow-up durations, and more diverse populations are needed to confirm the findings and evaluate long-term adherence and sustained benefits of such programs.”

OTHER INFORMATION

23) Registration number and name of trial registry

Does your paper address CONSORT subitem 23? *

Copy and paste relevant sections from the manuscript (include quotes in quotation marks "like this" to indicate direct quotes from your manuscript), or elaborate on this item by providing additional information not in the ms, or briefly explain why the item is not applicable/relevant for your study

”UMIN Unique trial Number: UMIN 000047789”

24) Where the full trial protocol can be accessed, if available

Does your paper address CONSORT subitem 24? *

Cite a Multimedia Appendix, other reference, or copy and paste relevant sections from the manuscript (include quotes in quotation marks "like this" to indicate direct quotes from your manuscript), or elaborate on this item by providing additional information not in the ms, or briefly explain why the item is not applicable/relevant for your study

”UMIN Unique trial Number: UMIN 000047789”

25) Sources of funding and other support (such as supply of drugs), role of funders

Does your paper address CONSORT subitem 25? *

Copy and paste relevant sections from the manuscript (include quotes in quotation marks "like this" to indicate direct quotes from your manuscript), or elaborate on this item by providing additional information not in the ms, or briefly explain why the item is not applicable/relevant for your study

”The authors did not receive support from any organisation for the submitted work.”

X27) Conflicts of Interest (not a CONSORT item)

X27-i) State the relation of the study team towards the system being evaluated

In addition to the usual declaration of interests (financial or otherwise), also state the relation of the study team towards the system being evaluated, i.e., state if the authors/evaluators are distinct from or identical with the developers/sponsors of the intervention.

	1	2	3	4	5	
subitem not at all important	○	○	○	○	◉	essential

選択を解除

Does your paper address subitem X27-i?

Copy and paste relevant sections from the manuscript (include quotes in quotation marks "like this" to indicate direct quotes from your manuscript), or elaborate on this item by providing additional information not in the ms, or briefly explain why the item is not applicable/relevant for your study

”R Nishio and T Dohi received research grants from M3 Inc., Tokyo, Japan. The other authors declare no conflicts of interest.”

About the CONSORT EHEALTH checklist

As a result of using this checklist, did you make changes in your manuscript? *

- ○ yes, major changes
- ○ yes, minor changes
- ◉ no

What were the most important changes you made as a result of using this checklist?

回答を入力

How much time did you spend on going through the checklist INCLUDING making changes in your manuscript *

Approximately 2 hours were spent reviewing the checklist and making corresponding changes in the manuscript.

As a result of using this checklist, do you think your manuscript has improved? *

- (selected) yes
- no
- その他:

Would you like to become involved in the CONSORT EHEALTH group?

This would involve for example becoming involved in participating in a workshop and writing an "Explanation and Elaboration" document

- (selected) yes
- no
- その他:

選択を解除

Any other comments or questions on CONSORT EHEALTH

回答を入力

STOP - Save this form as PDF before you click submit

To generate a record that you filled in this form, we recommend to generate a PDF of this page (on a Mac, simply select "print" and then select "print as PDF") before you submit it.

When you submit your (revised) paper to JMIR, please upload the PDF as supplementary file.

Don't worry if some text in the textboxes is cut off, as we still have the complete information in our database. Thank you!

Final step: Click submit !

Click submit so we have your answers in our database!

送信 フォームをクリア

Google フォームでパスワードを送信しないでください。

このフォームはドメイン外部で作成されました。 - 利用規約 - プライバシー ポリシー

このフォームが不審だと思われる場合 報告

Google フォーム
